# Saskemycin, a potent and selective antimycobacterial agent targeting a unique site on the ribosome

**DOI:** 10.21203/rs.3.rs-7820265/v1

**Published:** 2025-11-07

**Authors:** Gerard Wright, Michael Cook, Min Xu, Martino Morici, Dmitrii Travin, Wenliang Wang, Dorota Klepacki, Nandini Chhabra, Vishwas Rao, Henok Sahile, Dirk Hackenberger, Haaris Safdari, Max Berger, Martina Corazza, Austin Bond, Allison. Guitor, Dominique Tertigas, Lijun Wang, Adam Schaenzer, Linda Ejim, Venkateswarlu Yarlagadda, James Gomez, Michael Surette, Yossef Av-Gay, Neeraj Dhar, Deborah Hung, Nora Vázquez-Laslop, Alexander Mankin, Daniel Wilson

**Affiliations:** McMaster University; M.G. DeGroote Institute for Infectious Disease Research; McMaster University; University of Hamburg; University of Illinois at Chicago; McMaster University; University of Illinois at Chicago; University of Saskatchewan; Duke University; The University of British Columbia; McMaster University; MRC Laboratory of Molecular Biology; University of Hamburg; University of Hamburg; Broad Institute of Harvard and MIT; McMaster University; McMaster University; Institute of Microbiology, Chinese Academy of Science; McMaster University; Indian Institute of Technology; Broad Institute of MIT and Harvard; McMaster University; University of British Columbia; University of Saskatchewan; Broad Institute of MIT and Harvard; University of Illinois at Chicago; University of Illinois; University of Hamburg

## Abstract

Tuberculosis is the deadliest bacterial disease on the planet. The months-long regimen of multiple antibiotics required to treat tuberculosis profoundly affects the microbiome and leads to the development of antimicrobial resistance. Furthermore, non-tuberculous mycobacterial infections pose an increasing clinical challenge. Consequently, there is a growing need for new narrow-spectrum mycobacteria-targeting antibiotics with different mechanisms of action. Here, we report the discovery and characterization of a natural glycolipid antibiotic, saskemycin (SKM), which demonstrates potent and highly selective activity against mycobacteria. Genome sequencing, chemical analysis, and isotope feeding strategies reveal the unique structure and biosynthetic origin of SKM. SKM binds to the small ribosomal subunit at a site not targeted by any of the clinically relevant antibiotics acting on the ribosome. Bound to the ribosome, SKM corrupts the decoding center in a unique way, preventing stable binding of aminoacyl-tRNA in the A site and inhibiting translation in a sequence context-specific manner. Self-resistance in the producing organism is conferred by methylation of a single 16S rRNA nucleotide by SasO and SasN rRNA methyltransferases. These enzymes are orthologs of the ubiquitous RsmC and SpoU methyltransferases found in most bacterial genera but absent in mycobacteria, rationalizing SKM’s exquisite selectivity. The discovery of SKM provides an entry point for the development of selective, microbiome-sparing antimycobacterial antibiotics with a unique structure, binding site, and mechanism of action.

## Introduction

Tuberculosis (TB), caused by *Mycobacterium tuberculosis* (*Mtb*), is one of the most rampant infectious diseases worldwide, accounting for 1.3 million deaths annually, with an estimated one-fourth of the global population harboring a latent infection^1–3^. The combination of several *Mtb* features, such as a multilayered waxy cell wall, a facultative intracellular lifestyle, and the ability to establish long-term disease, makes TB one of the most challenging bacterial infections to treat. Infections caused by non-tuberculous mycobacteria (NTM) are also a growing concern. Slow-growing mycobacteria such as *M. ulcerans* and *M. avium*, along with faster-growing species like *M. fortuitum* and *M. abscessus*, present an increasing clinical challenge; the latter is particularly problematic in individuals with cystic fibrosis^4^.

Eradication of *Mtb* and NTM infections requires months-long treatment with multiple antibiotics^5,6^. In the case of TB, the standard treatment includes a combination of rifampin, isoniazid, pyrazinamide, and ethambutol for 8 weeks, followed by 18 weeks of rifampin and isoniazid^5^. For NTM like *M. abscessus*, treatment regimens include azithromycin paired with at least one other antibiotic for 12 months or more, and may ultimately require surgical intervention to resolve^7^. The prolonged antibiotic treatment during the course of the disease dramatically impacts the human microbiome, the healing process, and relapse (reviewed in^8,9^). Furthermore, lengthy antibiotic therapy facilitates the selection for antibiotic resistance that further exacerbates the treatment of these intrinsically recalcitrant infections, necessitating even longer therapies and additional drugs^5,10^. Therefore, new antibiotics selectively effective against *Mtb* and NTM must be discovered and developed. In particular, narrow-spectrum mycobacteria-targeting antibiotics that exert minimal effects on the host microbiome would be greatly beneficial.

Due to their impressive historical success, microbial natural products remain a promising source of new antibiotics^11^. *Streptomyces* species, in particular, are skillful producers of antimicrobial natural products. Indeed, the first clinically used antibiotic effective against *Mtb* was the *Streptomyces griseus*-produced streptomycin, a ribosome-targeting inhibitor of protein synthesis^12^. Although traditional phenotype-based screening methods commonly identify the already known antibiotics, genome mining in actinomycetes reveals that their biosynthetic potential is far from exhausted^13,14^.

Since the discovery of streptomycin, many other antibiotics have been identified that inhibit the growth of pathogenic bacteria by binding to one of the functionally important centers of the ribosome and interfering with translation^15–17^. However, because ribosome-targeting drugs often act on overlapping sites, some of the most prevalent resistance mechanisms can render bacteria simultaneously insensitive to different classes of antibiotics^18–20^. Therefore, discovering protein synthesis inhibitors that target novel ribosomal sites and arrest mycobacterial growth presents an exciting opportunity to avoid cross-resistance.

Here, we report the discovery of saskemycin (SKM), a unique cationic glycolipid antibiotic produced by *Streptomyces* sp. WAC00040. SKM binds to a distinct site on the ribosome that does not overlap with the binding sites of other clinically used antibiotics. SKM exhibits an unusual mechanism of action, preventing latching of the decoding center, thereby destabilizing binding of the aminoacyl-tRNA in the ribosomal A site. We demonstrate that dedicated rRNA methyltransferases protect the ribosome of the SKM producer from the harmful effects of the antibiotic. Importantly, modifications of the ribosome by related housekeeping RNA methyltransferases, which are found in many bacterial species but absent in mycobacteria, confer high-level resistance to SKM. These modifications account for the exquisite selectivity of SKM, which provides a new chemical scaffold for designing microbiome-friendly antimycobacterial drugs.

## Results

### A high-throughput screen of microbial extracts identifies a selective antimycobacterial compound with a peculiar structure

To identify highly selective antimycobacterial agents, we screened ~4,000 samples from the in-house microbial natural product extract library^21^ against *Mtb* H37Rv^22^ (**Fig. S1A**). Extracts from 40 strains significantly inhibited *Mtb* growth. We further tested the antimicrobial activity of extracts against the fast-growing mycobacterium *M. smegmatis*, as well as Gram-negative (*Escherichia coli*) and Gram-positive (*Staphylococcus aureus*) bacteria, and overlaid previous high-throughput screen data against the human HEK293 cell line to exclude toxic compounds^21,23^ (**Fig. S1B**). An extract from *Streptomyces* sp. WAC00040 (herein referred to as WAC40, originating from a farmer’s field north of Regina, Saskatchewan, Canada), was >1000-fold more potent than other candidate hits, with 0.001% (v/v) extract sufficient to fully inhibit the growth of *M. smegmatis* while showing neither activity against the other tested bacteria, nor significant toxicity against the human HEK293 cells (**Fig. S1B**).

**Figure 1:**
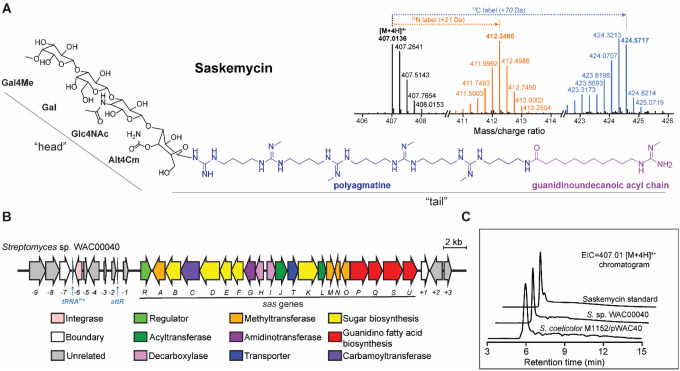
Identification of SKM, a highly specific antimicrobial natural product with a unique chemical scaffold. **A.** Structure of SKM. Elements of the structure are indicated and described in more detail in the main text. The ^13^C and ^15^N isotope labelling pattern of SKM is shown in the inset. The detected isotope pattern of SKM when culturing with ^13^C_6_-d-glucose or (^15^NH_4_)_2_SO_4_ as sole carbon or nitrogen source. Breaks in the x-axis indicate data from independent samples. Alt4Cm, 4-*O*-carbamoyl-L-altrose. **B.** SKM (sas) biosynthetic gene cluster. Genes are color-coded according to the predicted functions of the products. **C.** Heterologous production of SKM in *S. coelicolor*. The extracted ion chromatograms (EIC) of SKM ([M+4H]^4+^ = 407.01) are shown.

Fragmentation-based molecular network analysis^24^ of the active component secreted by WAC40 identified related chemical entities with a molecular mass range of 1160–1851 Da (**Fig. S2A**). The most abundant of these components, with a molecular mass of 1624.0247 Da, hereafter termed saskemycin (SKM), was purified (see Methods) and confirmed to be a highly potent antimycobacterial antibiotic, with a minimal inhibitory concentration (MIC) vs. *M. smegmatis* of 2 ng/mL. High-resolution electrospray ionization quadrupole time-of-flight mass spectrometry (HR-ESI-qTOF-MS) analysis identified SKM as a compound with a predicted molecular formula of C_70_H_137_N_21_O_22_, confirmed by stable isotope feeding experiments (Fig. 1A). The structure of SKM was determined using a combination of mass spectrometry (MS), tandem MS/MS, and nuclear magnetic resonance spectrometry (**Fig. S2-S12, Table S1**). SKM is a glycolipid antibiotic with a cobra-like structure composed of a tetra-saccharide ‘head’ comprised of 4-*O*-methyl-d-galactose, d-galactose, 4-*N*-acetyl-dglucosamine, and 4-*O*-carbamoyl-l-altrose units bridged through b-1,3-glycosyl bonds to a 2-*N*-methyl-polyagmatine acyl ‘tail’ (Fig. 1A). The tetra-saccharide is linked to the tail by a C-N glycosyl bond through C-1 of 4-*O*-carbamoyl-l-altrose. The polyagmatine acyl tail comprises five 2-*N*-methyl agmatine units end-capped by an 11-(2-methylguanidino)-undecanoic acyl chain. The unusual structure of SKM, unique amongst known antibiotics, prompted us to investigate its biosynthesis and mechanism of action.

### Identification of the biosynthetic gene cluster responsible for SKM production

Because of the peculiar structure of SKM, the conventional analysis^25^ of the WAC40 genome failed to locate its biosynthetic gene cluster (BGC). We then hypothesized that the *N*-methylagmatine groups in SKM could be derived from ω-*N*-methylarginine whose formation should be catalyzed by an arginine methyltransferase. Therefore, we used the sequence of the arginine methyltransferase SznE from the streptozotocin BGC^26,27^ to search the WAC40 genome for the putative SKM BGC. One low homology hit (31% identity to SznE) was found proximal to genes predicted to encode proteins with glycosyltransferase, acetyltransferase, carbamoyltransferase, aminotransferase, and amidinotransferase enzymatic activities anticipated to be present in the SKM BGC. The other adjacent genes encoded proteins possibly involved in furnishing the SKM tail and sugar modifications (Fig. 1B, **Table S2**). To verify that the identified gene cluster indeed represents the SKM BGC, a 36,452 bp DNA segment encompassing all of these genes was cloned into a plasmid and transformed into *Streptomyces coelicolor* for heterologous expression (**Fig. S13**). The HR-ESI-qTOF-MS analysis confirmed that the engineered *S. coelicolor* strain secreted SKM, thereby verifying that the cloned BGC is responsible for the production of SKM in the original producer strain WAC40 (Fig. 1C).

### SKM is a selective and effective antimycobacterial agent

**Figure 2:**
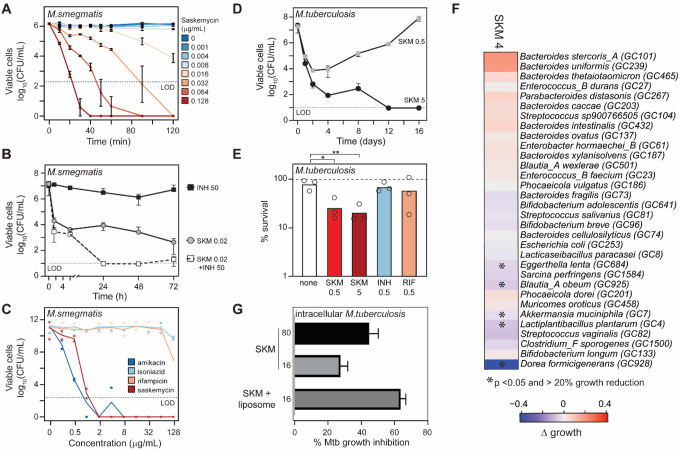
SKM is a selective and bactericidal microbiome-sparing antimycobacterial agent **A.** Time-kill assay with SKM in *M. smegmatis*. Points represent the average of two replicates and error bars represent standard error. LOD, limit of detection. **B.** Time-kill assay with SKM singly and in combination with isoniazid (INH) in *M.smegmatis*. Points represent the average of 2–3 biological replicates, and error bars represent standard deviation. Concentrations of each compound in mg/mL are indicated. **C.** Bactericidal activity in an induced dormancy model of *M. smegmatis*^29^. The line indicates the average of two replicate experiments. Individual data points are shown. **D.** Time-kill assay with SKM in *M. tuberculosis* Erdman. Points represent the average of 3 biological replicates, and error bars represent standard deviation. SKM concentration in mg/mL is indicated. **E.** Bactericidal activity in an induced dormancy model of *M.tuberculosis* Erdman^30^. Concentrations of SKM, rifampicin (RIF) and isoniazid (INH) in mg/mL are indicated. Survival, relative to day 0, was measured after 8 days of exposure. Individual data points are shown (n = 3). Significance was determined by two-tailed unpaired t-test (*, p = 0.0174; **, p = 0.0087). **F.** Activity of SKM against a panel of human microbiome strains. Growth relative to untreated control (OD_600_ treated – OD_600_ untreated) is represented in the heatmap. Growth was measured in triplicate. Asterisks indicate strains with a significant (p < 0.05) reduction in growth greater than 20%. **G.** THP-1-derived macrophages, infected with RFP-expressing *M. tuberculosis* H37Rv, were incubated for 3 days with different concentrations of SKM and liposome-formulated SKM. Mtb growth inhibition was calculated from the fluorescence signal increase. Results are normalized to bedaquiline (3 μM) positive and DMSO (1%) negative controls. Error bars represent standard error (SE), n = 4.

Having purified sufficient amounts of SKM from WAC40, we examined its antimycobacterial activity against a panel of fast and slow-growing mycobacteria. Consistent with its exceptional potency vs. *M. smegmatis*, the growth of all tested mycobacteria, except for *M. avium*, was arrested at sub-μg/mL concentrations of SKM (**Table S3**). Furthermore, SKM was bactericidal against *M. smegmatis* mc^2^155 with a minimum bactericidal concentration (MBC) of 0.032 μg/mL (Fig. 2A **and Fig. S14A**), 1000-fold lower than the reference bactericidal antibiotic amikacin^28^ (**Fig. S14B**), and its cidality was further enhanced when SKM was combined with isoniazid or rifampicin (Fig. 2B, **Fig. S14C**). Notably, in contrast to levofloxacin, isoniazid, or the antibacterial peptide LL-37, SKM was rapidly bactericidal (Fig. 2A, **Fig. S14B**). Given the indolent nature of mycobacterial infections, we examined whether the rapid bactericidal action of SKM would also manifest in models of dormancy^29^. Like amikacin, but in contrast to isoniazid or rifampicin, SKM retained activity against dormant *M. smegmatis* known to be resistant to most antimycobacterial compounds (Fig. 2C). The activity of SKM was generalizable to *M. tuberculosis* strains, with MBC values between 0.5–5 mg/mL depending on strain and inoculum used (Fig. 2D, **Fig. S14D**, and **Table S3**) and activity in a starvation-based model of dormancy (Fig. 2E)^30^.

The frequency of spontaneous SKM-resistance mutations in mycobacteria was low (4x MIC, 7×10^−10^; 8× MIC, 3×10^−10^ mutants/CFU). Only by serial passaging of *M. smegmatis* mc^2^155 in the presence of SKM were we able to isolate a resistant mutant (8-fold increase in MIC) that carried a mutation L59K in the lysine transferase LysX, an enzyme known to lysinylate phospholipids in mycobacteria (**Fig. S14E,F**)^31^. Subsequent analysis revealed that L59K is a gain-of-function mutation, likely leading to decreased SKM uptake (**Fig. S14F**). Given its promising antimycobacterial activity, we evaluated SKM against a panel of 32 Gram-positive and Gram-negative human microbiome strains (Fig. 2F). The majority of strains were insensitive to SKM, with only five showing >20% growth inhibition at 4 mg/mL SKM (p < 0.05), indicating promising narrow-spectrum activity. Consistent with our initial work with crude extracts, SKM showed no toxicity vs HEK293 cells (**Fig. S14G**).

The structure of SKM, particularly the polycationic tail, suggests that significant formulation development would likely be necessary for *in vivo* efficacy, especially for an intracellular pathogen like Mtb. We applied SKM to an intracellular infection model in macrophages. Consistent with expectations, free SKM showed only modest activity against intracellular Mtb; in contrast, encapsulation with HiPerFect transfection reagent significantly improved activity (Fig. 2G and **Fig. S15**), offering a strategy for future development of SKM as an intracellular antimycobacterial agent.

### SKM inhibits protein synthesis

The presence of the cationic polyagmatine tail could indicate that, analogous to cationic peptides^32^, SKM might disrupt the mycobacterial cell wall or membrane. We found, however, that SKM did not depolarize or permeabilize bacterial membranes (**Fig. S16**), making this mechanism of action unlikely. Thus, to identify the possible intracellular target of SKM, we employed the PROSPECT platform, where increased sensitivity to an inhibitor due to a decreased production of individual essential proteins can pinpoint a possible target^33^. The pattern of SKM-induced fitness changes of the PROSPECT *Mtb* strains, enriched for sensitivity in ribosomal protein hypomorphs, closely resembled those observed with ribosome-targeting inhibitors of translation tylosin and retapamulin (**Fig. S17**). These observations prompted us to examine the effect of SKM on protein synthesis.

**Figure 3:**
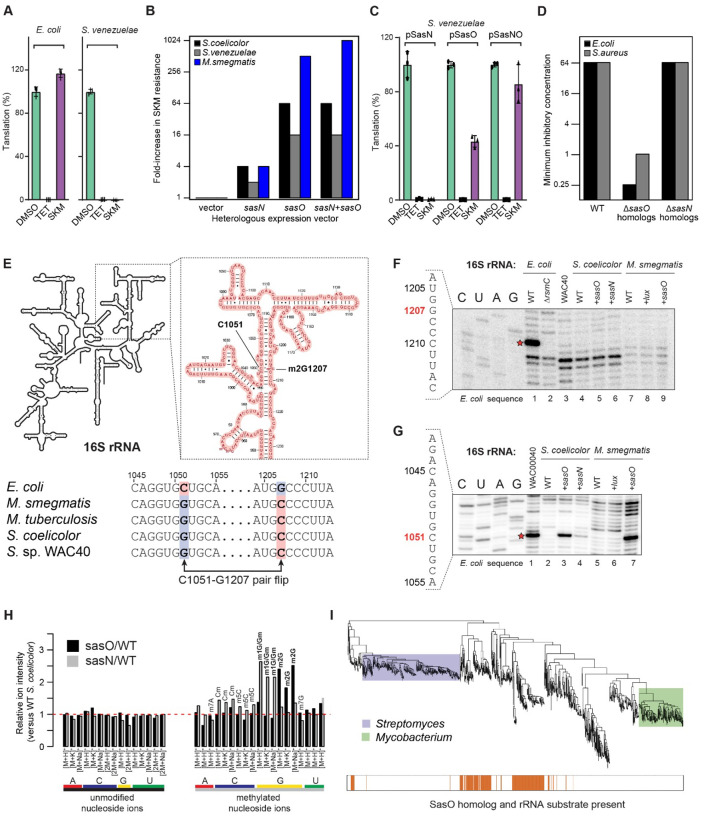
rRNA methyltransferases SasO and SasN provide self-resistance to SKM. **A.** Protein synthesis inhibition by SKM in S30 cell-free extracts from *E. coli* or *S. venezuelae*. Tetracycline (TET) was used as a comparator. Compounds were tested at 100 mM. Protein translation efficiencies were normalized to DMSO (no drug) control. Experiments were performed in triplicates and error bars represent standard deviations. Similar results were obtained from three independent experiments. **B.** Expression of SasO and SasN rRNA methyltransferases from *Streptomyces* sp. WAC40 confers resistance to SKM. **C.** Inhibition of cell-free translation by SKM in extracts prepared from *S. venezuelae* expressing none, SasN, SasO, or both methyltransferases. Experiments were performed as in **A**. **D.** Minimal inhibitory concentrations of SKM for *E. coli* and *S. aureus* strains with deletions of the homolog of *sasO* (*rsmC* in *E. coli* and SAUSA300_0526 in *S. aureus*) and of the sasN homolog (*lasT*in *E. coli* and *SAUSA300_0517* in *S. aureus*). **E.** (*top*) Location of the m2G1207-C1051 nucleotide pair in the secondary structure of *E. coli*16S rRNA. (*bottom*) The comparison of 16S rRNA sequences of the designated bacterial species shows the flip of this pair in actinobacteria including mycobacteria. **F.** Primer extension analysis of 16S rRNA nucleotides modifications in the position corresponding to G1207 (*E. coli* numbering). Note that this position (labelled with a red star) is modified in wt (*rsmC*-expressing) E. coli, but not in the Δ*rsmC* mutant or any of the tested *Streptomycesor Mycobacterium* strains wt or expressing *sasO*, *sasN* or *lux* [control] genes). **G.** Primer extension analysis showing the modification of nucleotide G1051 (labelled with a red star) in the 16S rRNA of SKM producer *Streptomyces* sp. WAC40 and the strains of *S. coelicolor* and M. smegmatis expressing *sasO*, *sasN*, but not *lux* (control) genes. **H.** Chemical nature of nucleotide modifications installed by SasO and SasN. Bar graphs of integrated ion intensities for individual ions for nucleosides and methylated nucleosides, expressed as a ratio relative to corresponding ions for the wt sample. Ions are annotated according to the molecular position of methylation, as defined by the retention time of individual standards. **I.** Representative phylogenetic tree of actinobacteria, illustrating the distribution of SasO homologs (orange lines in the rectangle). All the species carrying SasO homologs also have the G1051-C1207 base pair in the 16S rRNA that can be modified by SasO. SasO homologs are notably absent in *Mycobacteria* (green box) and most *Streptomyces* species (blue box), consistent with their SKM sensitivity.

SKM inhibited translation in a cell-free lysate of SKM-sensitive *Streptomyces venezuelae* with an IC_50_ of 0.41 ± 0.04 μM, similar to that of the well-characterized translation inhibitor tetracycline (0.60 ± 0.06 μM) (**Fig. S18**); *in vitro* protein synthesis was completely inhibited by 3.1 μM of SKM (Fig. 3A). Conversely, SKM was unable to inhibit protein synthesis in an *E. coli in vitro* translation system (Fig. 3A), consistent with the previously noted lack of inhibition of *E. coli* growth by SKM (**Fig. S1B**). These results suggest that the narrow selectivity of SKM’s antibacterial action may be related to differences in specific features of the translation apparatus in sensitive and resistant bacteria.

### Posttranscriptional rRNA modifications identify the SKM target and define its selectivity

The possibility that SKM interferes with bacterial growth by inhibiting the ribosome prompted us to evaluate potential self-resistance mechanisms in the native producer^34^. Two genes in the SKM BGC, *sasO* and *sasN*, are predicted to encode enzymes involved in post-transcriptional RNA modifications. SasO is an ortholog of the housekeeping rRNA methyltransferase RsmC, commonly found in many bacterial species but absent in mycobacteria (**Fig. S19**). SasN is similar to the rRNA/tRNA methyltransferases of the SPOUT family (**Fig. S20**). The *sasN* and *sasO* genes were cloned and expressed individually and in combination in SKM-sensitive *S. venezuelae* and *S.coelicolor* strains. The expression of *sasO* and, to a lesser extent, *sasN* conferred resistance to SKM (Fig. 3B, **Table S4**). Similar effects were observed when these genes were cloned and expressed in *M. smegmatis* (Fig. 3B, **Table S4**). The results of MIC testing (**Fig. S1B**) were further verified *in vitro* (Fig. 3C, **Fig. S18**). While SasN expression had little effect on SKM sensitivity of protein synthesis in *S. venezuelae* lysates (Fig. 3C, **Fig. S18A**), translation in cell-free extracts of *S. venezuelae* expressing SasO was far more resistant to SKM. Insensitivity of protein synthesis to SKM was further enhanced in lysates from cells expressing both SasO and SasN. In contrast, sensitivity towards tetracycline remained high in all the cell extracts tested (Fig. 3A,C, **Fig. S18B**).

The genomes of SKM-resistant *E. coli*, *S. aureus*, and many other intrinsically resistant strains do not carry the *sasO* gene, but encode instead SasO orthologs annotated as RsmC, the housekeeping rRNA methyltransferase that converts G1207 (*E. coli* numbering here and throughout) in 16S rRNA to 2-methyl guanosine (m2G). Inactivation of the *rsmC* gene in *E. coli* or its ortholog in *S. aureus* (SAUSA300_0526) increased sensitivity to SKM 256- and 32-fold, respectively, compared to the wild-type strains (Fig. 3D, **Table S4**), revealing the ribosome as a true SKM target and showing that RsmC-installed rRNA modification accounts for intrinsic SKM resistance.

In the SKM-resistant *E. coli* ribosomes, RsmC-targeted rRNA residue G1207 is base-paired with C1051. Interestingly, in the ribosomes of the SKM producer as well as in those from mycobacteria and SKM-sensitive streptomycetes, this base pair is flipped to G1051-C1207 (Fig. 3E). We wondered whether SasO and SasN target the same ribosomal site as RsmC, and if so, whether they modify C1207 or G1051. Therefore, we sought to map the site(s) and the nature of the modifications introduced in the rRNA by SasO and SasN. The RsmC-mediated methylation of G1207 to m2G in *E. coli* 16S rRNA could be readily detected by primer extension (Fig. 3F, lane 1). As expected, the band corresponding to methylated G1207 was absent when primer extension was carried out with the rRNA from D*rsmC E. coli* cells (Fig. 3F, lane 2). Curiously, the same band was also absent when primer extension was carried out on 16S rRNA from SasO/SasN expressing strains of WAC40, *S. coelicolor*, and *M. smegmatis* (Fig. 3F, lanes 3–9). The primer extension results were corroborated by direct Nanopore sequencing of the 16S rRNA from *S. coelicolor*, which showed the presence of an unmodified cytosine at position 1207 (**Fig. S21A**). Thus, it became clear that SasO and SasN must be acting on a different rRNA site.

Nanopore sequencing indicated the presence of an unusual nucleotide at position 1051, encoded as G, in the 16S rRNA gene of *S. coelicolor* and other Actinobacteria (**Fig. S21A,B**). Consistent with these findings, a specific band on the primer extension gel revealed a potential modification at G1051 of 16S rRNA from the SasO-expressing *S. coelicolor* and *M. smegmatis* strains, but not in the parental strains (Fig. 3G, lanes 2–3 and 5–7). A band corresponding to the modified G1051 of the 16S rRNA of the native SKM producer WAC40 was also detected by primer extension (Fig. 3G, lane 1). Notably, a weaker band corresponding to the same position appeared when primer extension was carried out on the 16S rRNA from the *S. coelicolor* strain expressing SasN (Fig. 3G, lane 4). Together with the data from microbiological and *in vitro* testing of the SasO and SasN expressing strains (Fig. 3B–C), these results strongly argue that in SKM-producing bacteria, SasO and, to a lesser extent, SasN confer self-resistance to SKM by catalyzing post-transcriptional modification of G1051 in 16S rRNA, thereby protecting the producer’s ribosomes from SKM action.

To determine the nature of the chemical modifications introduced by SasO and SasN, we carried out the LC-MS analysis of the nucleoside composition of the 16S rRNA isolated from SasO- or SasN-expressing *S. coelicolor*. Expression ofSasO increased the abundance of ions corresponding to m2G compared to the control strain (Fig. 3H**, Fig. S21C**). This finding, along with the results of primer extension and Nanopore sequencing, is consistent with SasO conferring SKM resistance by mono-methylating N2 of G1051 in the 16S rRNA. The 16S rRNA isolated from the SasN-expressing strain exhibited an increased amount of a differently modified guanosine, carrying a methyl group either at the N1 of the nitrogen base (m1G) or the 2’ hydroxyl of the ribose (Gm). Because SasN shares common structural motifs and catalytic residues with ribose 2’-OH methyltransferases from the SpoU-TrmD (SPOUT) methyltransferase family (**Fig. S20**), SasN likely methylates the 2’OH of G1051 ribose, converting it to Gm1051.

Analysis of sequenced bacterial genomes (Fig. 3I**, Fig. S19**) revealed a strong association between the presence of RsmC homologs in the genomes of proteobacteria, a large diverse phylum of Gram-negative bacteria, and the occurrence of the C1051-G1207 base pair in their 16S rRNA. In contrast, firmicutes, a phylum of predominantly Gram-positive bacteria, including *S. aureus*, that carry 16S rRNA with the flipped base pair (G1051-C1207) frequently harbor the SasO-like RNA methyltransferase genes. Only limited groups of bacteria, including mycobacteria and streptomycetes, lack either methyltransferases, consistent with their sensitivity to SKM. Thus, genome mining, combined with the results of our biochemical and genetic experiments, demonstrates that the strikingly narrow selectivity of SKM action is driven by the idiosyncratic patterns of rRNA modifications, which leave only a small fraction of bacterial species susceptible to inhibition by this antibiotic.

### SKM binds to the ribosome at a unique site

**Figure 4:**
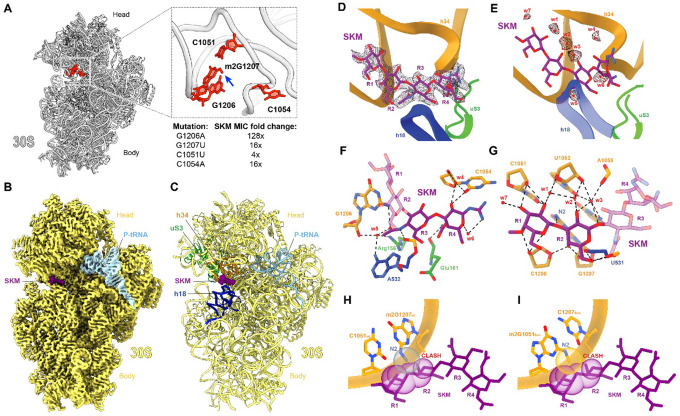
SKM binding site on the ribosome. **A.** Location of 16S rRNA nucleotides (shown in red) substituted in the selected SKM-resistant *E. coli* mutants. The blue arrow points to the N2-methylation of G1207 installed by RsmC in *E. coli*. **B.** Cryo-EM map of the 30S subunit (yellow) of SKM-stalled ribosome (state 1) with P-tRNA (cyan) and SKM (purple). **C.** Molecular model for State 1 from (**B**), with 30S (yellow), P-tRNA (cyan), SKM (purple), h18 (blue), h34 (gold), and uS3 (green). **D.** Cryo-EM density (mesh) and model (purple) for SKM. **E.** Cryo-EM density (mesh) for water molecules within the SKM (purple) binding pocket. **F-G.** Direct and water-mediated hydrogen bond interactions (dashed lines) of SKM (purple) within the binding pocket. **H-I.** Steric clashes predicted between SKM and methyl group (grey) of (**H**) m2G1207 of wild-type (wt) *E. coli* ribosome (PDB ID: 7K00)^38^ and of (**I**) m2G1051 of an *in silico* modelled *S. coelicolor* ribosome (Sco).

The location of the resistance modifications in the 16S rRNA indicated the likely site of SKM action on the ribosome. To better understand the ribosomal elements involved in interactions with the SKM pharmacophore, we isolated rRNA mutations that confer SKM resistance. To this end, we engineered an SKM-sensitive *E. coli* strain that lacks *rsmC* and carries a single rRNA operon on its chromosome, thereby allowing for the selection of resistance mutations in rRNA genes^35,36^. When these cells were plated on LB agar supplemented with 32×MIC of SKM, resistant mutants appeared with a frequency of ~10^−7^. Sequencing the 16S rRNA gene in several resistant strains revealed the presence of mutations G1206A, G1207U, C1051U, or C1054A (Fig. 4A**, Table S4**). The constellation of the sites of the mutations and the modifications induced by the resistance-conferring rRNA methyltransferases delineated the location of the SKM binding site in the ‘head’ of the small ribosomal subunit.

To determine the atomic interactions of SKM with its target and gain insights into the mechanism of action, we solved the structure of the translating ribosome bound to SKM. For these studies, *E. coli* ribosomes were isolated from the SKM-susceptible D*rsmC* strain and were confirmed to be susceptible to SKM in an *in vitro* translation system (**Fig. S22A**). These ribosomes were then used to translate a model mRNA encoding the short peptide Met-Leu-Ile-Phe^37^ in the presence of 50 mM SKM (**Fig. S22B**). The SKM-stalled ribosome complexes (SKM-SRCs) were subjected to single-particle cryo-electron microscopic (cryo-EM) analysis. *In silico* sorting of the particles revealed seven distinct functional states of the ribosome (**Fig. S23**), with the highest populated state (State 1, 37%) containing P-tRNA, but no A-tRNA (Fig. 4B). When this state was refined to 2.3 Å resolution (**Fig. S24, Table S5**), we observed additional density attributable to SKM bound between 16S rRNA helices h18 in the body and h34 in the head of the 30S subunit (Fig. 4B–D) – at the site consistent with the location of the resistance mutations and RsmC/SasO/SasN rRNA modifications (Fig. 4A). After combining additional functional states and performing a focused refinement on the 30S subunit, the quality of the cryo-EM density for SKM allowed an unambiguous *de novo* modelling of the tetra saccharide rings – the ‘head’ of the SKM cobra-like structure (Fig. 4D). At the distal end of SKM, rings R3 and R4 interact with the 16S rRNA residues A532 of the small ribosomal subunit body, and G1206 and C1054 in the head, as well as with Arg156 and Glu161 of the ribosomal protein S3 (Fig. 4F). However, the majority of interactions with the ribosome involve rings R1 and R2 of SKM, that insert into the minor groove of 16S rRNA helix h34, establishing a network of hydrogen bonds with nucleotides G1207-C1208 in one RNA strand and C1051-U1052/A1055 in the other (Fig. 4G). Additional density for seven water molecules in proximity of SKM was observed (Fig. 4E), five of which (w1-w5) facilitate indirect interactions between SKM and the 30S subunit (Fig. 4F,G). Notably, the oxygen atom that links rings R1 and R2 of SKM is within hydrogen bond distance (3.1 Å) to the N2 of G1207 (Fig. 4G), the site of RsmC-mediated methylation that confers resistance to SKM (Fig. 4A). Aligning the structure of the SKM-bound ribosome with that of an *E. coli* ribosome from a wild-type *rsmC*-containing strain^38^ reveals that methylation of the N2 of G1207 would lead to steric clashes with R1 and R2 of SKM (Fig. 4H), thereby providing the structural explanation for RsmC-mediated SKM resistance. Similarly, *in silico* modeling of the SasO-methylated G1051 indicates that SKM resistance is also likely due to a direct steric clash of the inhibitor with the N2 methyl group appended to the guanine base (Fig. 4I).

### SKM stabilizes an open state of the small ribosomal subunit and inhibits stable binding of the tRNA in the A site

**Figure 5:**
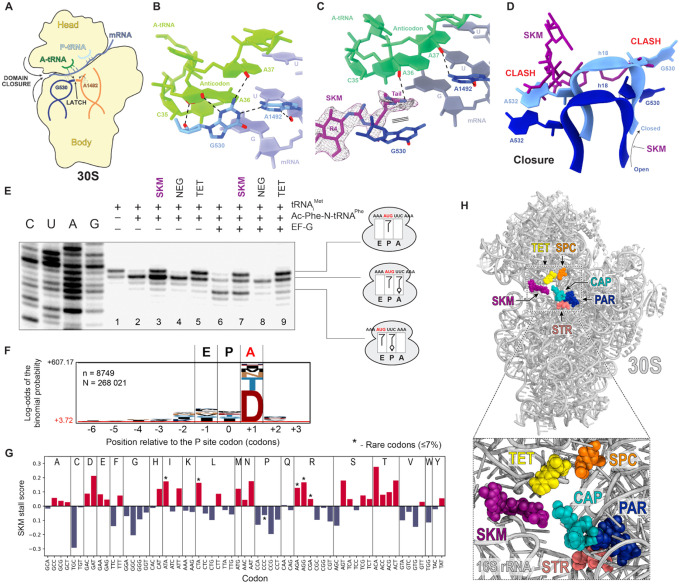
Mechanism of translation inhibition by SKM. **A.** Schematic representation of domain closure (and latching of G530 in h18 with A1492 in h44) upon decoding of the mRNA (blue) by the A-site tRNA(green). **B.** Closed and latched State 4 ribosome (PDB ID 5UYM)^39^, showing the base-pairing between the A-tRNA anticodon (light green) and the mRNA codon (indigo) with the domain closure achieved by latching between G530 and A1492 (light blue), which leads to displacement of SKM. **C.** Same view as B, but with State 3. Cryo-EM density (mesh) for tail of SKM from State 3 stacking on G530 of h18 (blue) and forming a potential hydrogenbond (black dashed line) with the 2´-OH of A36 of the A-tRNA anticodon (dark green). **D.** Superimposition of State 3 with State 4 (PDB ID 5UYM)^39^, showing steric clashes between SKM (purple) of State 3 and the loop of h18 of State 4 (light blue). h18 of State 3 is shown in dark blue. **E.** SKM inhibits A-site tRNA accommodation *in vitro*. Compare lanes 2 and 3 and note the reduced one nucleotide shift reflecting the reduced binding of N-acetyl-Phe-tRNA^Phe^ to the model mRNA-ribosome-tRNA_i_^Met^ complex in the presence of SKM. A similar effect is observed in the presence of tetracycline (TET) that sterically blocks tRNA binding in the A site^49,50^, but not negamycin (NEG), an inhibitor of translocation^47^. **F.** pLogo analysis^115^ of the amino acid sequences at the preferential sites of SKM-induced ribosome stalling in the antibiotic-treated *E. coli* cells deduced from Ribo-seq analysis. **G.** The change of the A-site codons occurrence at the sites of preferential ribosome stalling in SKM-treated cells deduced from Ribo-seq analysis. Higher stalling scores reflect the increased occupancy of the codons in the cells treated with the antibiotic Rare *E. coli* codons (encoding less than 7% of all instances of the corresponding amino acid) are marked with asterisks. **H.** Superposition of the SKM binding site with the sites of binding of the major classes of clinically relevant antibiotics targeting the small ribosomal subunit. Overview (top) and close-up views (bottom) of the ribosome-bound SKM (purple) relative to tetracycline (TET, yellow), aminoglycosides streptomycin (STR, light red) and paromomycin (PAR, dark blue), tuberactinomycin capreomycin (CAP), and aminocyclitol spectinomycin (SPC).

While State 1 revealed the location of the SKM binding site and its atomic interactions with the ribosome, it did not immediately explain the mechanism of SKM action. However, several other well-resolved states yielded important insights into the mode of translation inhibition by SKM. Normal decoding of the A-site mRNA codon during aminoacyl-tRNA delivery triggers “latching” of the decoding center due to the interaction of G530 in h18 with A1492 in h44, and rearrangement of the 30S subunit architecture described as “domain closure”^39,40^ (Fig 5A). We observe this conformation in State 4 (7% of the particles, 2.8 Å resolution) representing SKM-free ribosomes with P-tRNA and an accommodated A-tRNA^39^ (Fig. 5B). A very different picture is observed in the SKM-bound ribosomes (State 2 and 3): In State 2 (33% of the particles), density was observed for elongation factor Tu (EF-Tu) caught in the act of delivering tRNA to the A-site of the ribosome (**Fig. S23**). However, when this population was refined to 2.3 Å, the density for EF-Tu and A-tRNA became weaker and more fragmented, suggesting that SKM may interfere with the delivery of the A-site tRNA, likely resulting in multiple states of the ternary complex on the ribosome^39^. Importantly, in State 2, SKM stabilizes an open conformation of the 30S subunit that precludes latching and therefore is likely to be unfavorable for accepting the incoming A-site tRNA. In State 3 (11% of the particles, 2.6 Å resolution, **Fig. S24, Table S5**), the SKM-bound ribosome is also observed in the open conformation, despite the presence of an accommodated A-tRNA. Here, an additional density is seen extending from ring R4 of SKM, enabling partial modeling of the SKM-tail (Fig. 5C). The tail appears partially ordered due to stacking interactions with G530 of the 16S rRNA in the *syn* conformation, characteristic of the open state of the 30S subunit^39^, as well as a potential hydrogen bond with A36 in the anticodon of the A-tRNA (Fig. 5C). Thus, due to the steric clash between SKM and h18 in the small subunit closed conformation, SKM favors an open unlatched state of the decoding center likely preventing stable binding of aminoacyl-tRNA in the A site (Fig. 5D).

In SKM-bound States 1–3, the ribosome was observed in a non-rotated state with peptidyl-tRNAs interacting with the P sites of the small and large subunits (the P/P position) (**Fig. S23**). However, in State 5 (13%, 2.6 Å, **Figs. S23, Table S5**), the SKM-bound ribosome has undergone the intersubunit rotation typical of the first phase of translocation; the anticodons of the tRNAs are still bound in the P and A sites on the small subunit, while their acceptor ends shifted to the E and P sites, respectively, on the large subunit (the A/P and P/E positions)^41^ (**Fig. S25A-C**). Unexpectedly, the rotated SKM-bound 30S subunit still remains in an open conformation (**Fig. S25D-F**). While the SKM-bound ribosome is able to attain the hybrid state, the subsequent steps of the translocation reaction are probably hindered since no late translocation states were present in the dataset (**Fig. S23**), as we observed previously for the translocation inhibitor PamB2^42^. Thus, we hypothesize that by interacting with the subunit head and body, SKM may also prevent head-swiveling that accompanies the movement of the mRNAs and tRNAs through the ribosome at the late translocation steps (**Fig. S25G-I**)^43–46^.

### The mechanism of translation inhibition by SKM

The cryo-EM structural data suggested that SKM interferes with the binding of tRNA in the ribosomal A-site and could potentially affect translocation. To experimentally test the mechanism of SKM action, we monitored tRNA binding and EF-G-catalyzed ribosome translocation by toeprinting analysis^47,48^. The binding of deacylated tRNA_i_^Met^ in the P-site positions the ribosome at the AUG codon of a model mRNA (Fig. 5E, lane 1). The subsequent binding of N-acetyl-Phe-tRNA^Phe^ in the A-site converts the ribosome to the pre-translocation state and shifts the toeprint band by one nucleotide (Fig. 5E, lane 2). Notably, the addition of SKM to this complex partially restored the toeprinting pattern characteristic of the ribosome carrying only the P-site tRNA, supporting the structural analysis inference that SKM interferes with the stable binding of A-site tRNA (Fig. 5E, lane 3). A similar but stronger effect was noted in the presence of tetracycline (TET), an antibiotic that directly blocks tRNA binding in the A-site by steric hindrance with the anticodon^49,50^ (Fig. 5E, lane 5). Upon the addition of EF-G, the drug-free ribosomes translocate to the next codon (Fig. 5E, lane 6), but in the sample containing SKM, the majority of ribosomes remained associated with the start codon (Fig. 5E, lane 7). The incomplete translocation can result from direct interference of SKM with this process, as in the case of the control antibiotic negamycin (NEG), a translocation inhibitor that stabilizes the A-site tRNA^47,51^ (Fig. 5E, lane 8). Alternatively, it could be an indirect consequence of the SKM-mediated A-site tRNA displacement, as illustrated by the control TET-containing sample (Fig. 5E, lane 9).

SKM interference with decoding center latching and putative interaction of the SKM tail with the A-site tRNA anticodon (Fig. 5A) may differentially affect binding of individual tRNAs to the A site. Therefore, we wondered whether, similar to other ribosome-targeting antibiotics^42,52^, SKM would inhibit translation in a context-specific manner. We used ribosome profiling (Ribo-seq) to analyze the effect of SKM on the progression of ribosomes along mRNAs in bacterial cells^53^. After a brief treatment of SKM-sensitive *E. coli* cells with 25×MIC of the antibiotic, the ribosome-protected mRNA fragments were isolated and sequenced, and the ribosome occupancy of individual mRNA locations was compared to that in untreated cells (**Fig. S26**). Analysis of the amino acids encoded in mRNA at the sites of SKM-induced translation arrest reveals that SKM preferentially stalls the ribosome when tRNAs delivering Asp or Thr are accommodating into the A site (Fig. 5F,G). Examination of the A-site mRNA codons indicates that indeed, those decoded by tRNA^Asp^ and tRNA^Thr^, but also specific codons decoded by other tRNAs (tRNA^Asn^, tRNA^Met^, tRNA^Glu^), were enriched in the A site of SKM-stalled ribosomes (Fig. 5G). Although no common features emerge upon inspecting the respective tRNAs, it is possible that either a direct interaction between the SKM tail and the anticodon stem-loop of the tRNA, or a lower intrinsic affinity of these tRNAs for the ribosome with the unlatched decoding center, accounts for the context specificity of SKM action.

## Discussion

We have discovered a unique natural antibiotic that is highly selective against mycobacteria, including *Mtb* and non-tubercular mycobacterial pathogens. The discovery of SKM re-emphasizes the importance of phenotypic screens of *Streptomyces* natural product extracts, which proved so effective in the past and can still identify exciting new agents with potential for drug development. Identification of SKM was possible due to the nature of the screening platform, which was designed to specifically identify antibiotics capable of inhibiting *Mtb* while sparing common Gram-positive and Gram-negative bacterial species. Our biochemical and genetic analyses showed that the production of SKM is driven by a distinctive and unusual BGC that is difficult to identify using conventional genome mining algorithms, emphasizing the ongoing importance of empirical approaches in antibiotic discovery.

Like many antibiotics, SKM targets the ribosome. However, SKM acts on a ribosomal functional site that is not exploited by any current medically used antibacterials that bind the small ribosomal subunit, such as aminoglycosides, aminocyclitols, tetracyclines, or capreomycin (Fig. 5H). SKM’s binding site only marginally overlaps with the sites used by non-clinical antibiotics odilorhabdins^54^ and lariocidin^55^ (**Fig. S27**).

Due to its unusual structure and unconventional binding, SKM shows a mode of action not seen with other antibiotics (**Fig. S28**). Unlike streptomycin, other aminoglycosides, odilorhabdins, negamycin, and possibly lariocidin, which enhance the accommodation of non-cognate aminoacyl-tRNAs, and unlike tetracycline, which directly clashes with the A-site tRNA, SKM influences decoding by destabilizing A-site tRNA binding. It does this by preventing the latching of the decoding center and keeping the small subunit in an open conformation (Fig. 5A–D). Interestingly, even with the 30S subunit remaining in an open state (States 1–3 and 5 of the cryo-EM reconstructions), the SKM-bound ribosome can occasionally accommodate aminoacyl-tRNA in the A site, form a peptide bond, and even initiate translocation advancing to the rotated state (**Fig. S28**). However, it is likely unable to complete the translocation cycle because SKM immobilizes parts of the small subunit, which need to have a sufficient degree of mobility for successful translocation (**Fig. S25**). The uniqueness of SKM is underscored further by the idiosyncratic context-specificity of its action. SKM preferentially stalls the ribosome when specific mRNA codons are decoded in the A site (Fig. 5G). Since SKM does not directly interact with the mRNA or the newly forming peptide, its context specificity probably depends on tRNA properties. The retention of tRNA in the A site of the unlatched decoding center might rely on unique features of the tRNA structure. However, we cannot rule out the possibility that the tail of SKM, which remains mostly unresolved in our cryo-EM reconstructions, may make direct contact with the tRNA anticodon, providing an alternative or possibly complementary explanation for the observed context specificity.

Perhaps the most notable feature of SKM is its remarkable selectivity. While serving as a highly effective bactericidal antimycobacterial agent, SKM is not toxic to mammalian cells and, importantly, does not inhibit the growth of many other bacterial species. Furthermore, a screen of human microbiome isolates showed minimal impact of SKM on growth, which is promising for an antibacterial agent that would largely spare the microbiome during clinical use. Our findings identified a key factor that accounts for SKM’s selectivity within bacterial species. The extraordinarily narrow spectrum of the SKM action is determined by the lack of the common posttranscriptional rRNA modifications in ribosomal binding site of the sensitive species. The RsmC or SasO/SasN rRNA methyltransferases make the ribosome resistant to SKM, and the absence of these rRNA modifiers in mycobacteria and very few other bacteria accounts for SKM’s high selectivity. Most proteobacteria have the C1051-G1207 base pair in their 16S rRNA and possess RsmC-like rRNA methyltransferases that methylate G1207 to m2G. While in the firmicutes, this base pair is inverted, resistance to SKM is accounted for by SasO-like methyltransferases that modify G1051 to m2G on the opposite strand of the rRNA stem. Any of these modifications creates a steric clash with SKM, preventing the antibiotic binding and rendering the ribosome highly resistant. It is the lack of orthologs of any of these methyltransferases that renders mycobacteria sensitive to SKM. In the SKM producer, SasO provides a high degree of resistance, which can be further enhanced by SasN.

The peculiar cobra-like chemical structure of SKM and the unusual nature of its BGC call for their further exploration. To determine the role of the individual body parts of the SKM ‘cobra’ in biological activity, direct chemical synthesis or BGC engineering will be necessary. While the ‘head’ of the cobra structure of SKM was clearly defined in its binding site, the tail was mostly invisible, indicating its flexibility. Nevertheless, the presence of genes responsible for tail assembly within the SKM BGC suggests its significance. The tail could possibly account for the context specificity of SKM action. Alternatively, or complementary, the tail could facilitate SKM uptake. Both the head and the tail of SKM offer many interesting routes for further improvement of the pharmacological properties of SKM.

In conclusion, SKM represents a unique chemical structure produced by a BGC that defies identification by current algorithms. It is extremely potent against mycobacteria, spares most tested human microbiome species, and has a novel mechanism of action with a unique binding site on the bacterial ribosome. It offers a unique entry point for a route towards the development of a new antimycobacterial antibiotic that would expand our arsenal of weapons against the most deadly bacterial pathogens.

## Methods

### Strains and culture conditions

Strains used in this study are summarized in **Table S6**. *E. coli* strains were cultured in LB broth Lennox (Bioshop) at 37°C with shaking at 250 rpm. *S. cerevisiae* VL6–48N was grown in yeast extract-peptone-dextrose (YPD) medium (yeast extract 10 g/L, peptone 20 g/L, dextrose 20 g/L) supplemented with adenine (100 mg/L) at 30 °C for spheroplast preparation. Yeast transformants^56^ were selected on sorbitol-dextrose medium without tryptophan (sorbitol 180 g/L, glucose 20 g/L, agar 20 g/L, 880 mL ddH_2_O, SD-Trp). 10× yeast nitrogen base (100 mL): yeast nitrogen base w/o amino acids and ammonium sulfate, 1.7 g; (NH_4_)_2_SO_4_, 5 g; amino acid mix w/o tryptophan, 0.832 g; 100× adenine (10 mg/mL); and 100× 5-Fluoroorotic acid (100 mg/mL) were added into SD-Trp medium after autoclaving. Positive yeast transformants were grown in SD-Trp liquid selective medium (sorbitol 180 g/L, glucose 20 g/L, 890 mL ddH_2_O. 10× yeast nitrogen base and 100× adenine were added after autoclaving) for plasmid isolation. *Streptomyces* strains were grown in tryptic soy broth-yeast extract medium (tryptic soy broth 30 g/L, yeast extract 5 g/L, TSBY) at 30 °C, 250 rpm for genomic DNA isolation and seed culture preparation, and on soy flourmannitol medium (soy flour 20 g/L, d-mannitol 20 g/L, agar 20 g/L, pH 7.2–7.4, SFM) at 30 °C for sporulation and conjugation (supplemented with 20 mM MgCl_2_). Fermentation was performed in Bennett’s medium (potato starch 10 g/L, casamino acids 2 g/L, yeast extract 1.8 g/L, Czapek mineral mix 2 mL, pH 6.8; Czapek mineral mix: KCl 10 g, MgSO_4_·7H_2_O 10 g, NaNO_3_ 12 g, FeSO_4_·7H_2_O 0.2 g, concentrated HCl 0.2 mL, ddH_2_O 100 mL, filter sterilize before use) at 30 °C, 250 rpm for 6 d. Antibiotics were supplemented as required for selection (ampicillin 100 μg/mL, kanamycin 50 μg/mL, nalidixic acid 25 μg/mL, trimethoprim 50 μg/mL, hygromycin 50 μg/mL [*Streptomyces*] and 150 μg/mL [*E. coli*]).

### Mycobacterial strains

*M. tuberculosis* H37Rv harbouring an RFP-expressing hygromycin resistant pTEC27 plasmid were utilized for macrophage infection models^57^. The bacteria were routinely grown in 7H9 broth (Difco Middlebrook) supplemented with 10% (v/v) OADC (5% bovine albumin fraction, 2% dextrose, 0.004% catalase, 0.05% oleic acid and 0.8% sodium chloride solution) and 0.05% (v/v) Tween-80 (Sigma-Aldrich) at 37 °C in standing cultures. Hygromycin B was added to the medium at a final concentration of 50 μg/mL. All mycobacterial strains were grown at 37 °C, with the exception of *M. ulcerans*, which was maintained at 30 °C. Mycobacterial strains were grown in standard media, Middlebrook 7H9 (Becton Dickinson) containing 0.2% glycerol, 10% OADC, and 0.05% Tween-80 in standing cultures. *M. smegmatis* was grown in shaking cultures at 250 rpm.

### Other bacterial strains

*Corynebacterium* spp. were grown in tryptic soy broth and on tryptic soy agar at 37 °C. *Nocardia* sp. WAC07162 and *Tsukamurella* sp.WAC06889b were grown on Bennett’s agar at 30 °C; for MIC assays, they were grown in tryptic soy broth at 30 °C. *Rhodococcus equi* ATCC 14887 was grown on brain heart infusion agar at 30 °C; for MIC assays, it was grown in tryptic soy broth at 30 °C.

### High-throughput screen

The *M. tuberculosis* screen was performed as described^22^. Briefly, *M. tuberculosis* H37Rv pUV3583c:GFP was inoculated from frozen culture into a roller bottle containing 100 mL of Middlebrook 7H9 media supplemented with 10% OADC, 0.05% Tween-80, and 10 mM sodium acetate and grown at 37 °C with rolling for 5 days until OD_600_ reached ~ 0.8. The OD_600_ was adjusted to 0.025 into acetate containing 7H9 media. A culture of 50 mL and 0.5 mL of NP extract were added in duplicate to 384-well assay plates using a Bravo liquid handler (Agilent). Assay plates were incubated in sealed containers at 37 °C for 4 days before reading GFP fluorescence. Duplicate data from screens were converted to composite Z scores by cosine correlation, using DMSO controls as reference^58^. The composite Z-score threshold for hits was selected relative to the Z-scores of rifampicin that gave a Z’-factor of 0, where the distance separating the positive and negative controls is 3× the sum of the standard deviations of the two populations^22^.

### Antibiotic susceptibility testing

*S. aureus* and *E. coli* strains were grown in Mueller Hinton II Broth (MHB) (cation adjusted) (Becton Dickinson), at 37 °C with aeration for 18 h. *Streptomyces* strains were grown in TSBY medium at 30 °C for 24 h for *S. venezuelae* or 48 h for S. coelicolor. Susceptibility testing was performed using the microdilution broth method, with inoculum prepared using the colony suspension method, according to CLSI guidelines^59^. Reported data are the averages from at least two experiments. Mycobacterial strains were cultured in standard 7H9 media, Middlebrook 7H9 containing 0.2% glycerol, 10% OADC, and 0.05% Tween-80, and diluted to a final CFU/mL of ~5 × 10^5^ cells/mL. CFU totals were confirmed to be within the range of 3–8 × 10^5^ cells/mL by plating of dilutions of the inoculum on Middlebrook 7H10 (Becton Dickinson) agar, containing 0.5% glycerol and 10% OADC. Susceptibility was assessed at 2–3 days (*M. smegmatis, M. fortuitum, M. abscessus*), 7 days (*M. tuberculosis* H37Ra*, M. bovis, M. avium*), or 14 days (*M. ulcerans*). Reported data are the averages from at least two experiments. Mycobactericidal concentrations were determined by plating dilutions of *M. smegmatis* or *M. tuberculosis* on 7H10 agar before and after treatment with SKM for 2 or 7 days, respectively. Reported data are the averages from at least three experiments.

### Genome sequencing and assembly

Genomic DNA extraction and Illumina sequencing of WAC40 were carried out as previously described^60^. gDNA from WAC40 was prepared for Illumina Sequencing (MiSeq 2 × 250 bp reads) using the Next Ultra II kit (New England Biosciences) with 444 ng input DNA (sonicated to 600 bp via Covaris MicroTUBE) and a double size selection with purification beads. Sequencing was performed by the McMaster Genomics Facility in the Farncombe Institute at McMaster University (Hamilton, ON, Canada). Sequencing reads were trimmed using skewer v0.2.2^61^ (-q 25 and -Q25) and merged using FLASH v1.2.11 with default parameters^62^.

High molecular weight gDNA of WAC40 was isolated using the salting out procedure^63^ followed by removing the RNA by RNase A treatment. For Nanopore sequencing of WAC40, 450 ng of high-molecular weight gDNA was prepared using the Rapid Barcoding Kit (SQK-RBK004) from Oxford Nanopore Technologies. This sample was pooled at equal volumes with 4 other genomes and sequenced on a MinION R9.4.1 flow cell for 48 h. Read traces were classified using Deepbinner v0.2.0^64^ before basecalling with ONT’s Guppy basecaller (2.3.1; dna_r9.4.1_450bps configuration). Reads were binned using Deepbinner v0.2.0^64^ then trimmed using Porechop v0.2.4 (https://github.com/rrwick/Porechop). Trimmed and merged Illumina reads from previous Illumina sequencing along with trimmed long reads were *de novo* assembled using Unicycler v0.4.8b^65^ using Pilon v1.23^66^ and SPAdes v3.13.0^67^.

The hybrid assembly sequence for WAC40 is available from BioProject PRJNA804892.

### DNA cloning

Plasmids are summarized in **Table S6**. Primers used in this work are listed in **Table S7**. Plasmid pMV306hsp+LuxG13^68^ was modified to remove a *Sap*I site within the *luxA* gene, remove the hsp promoter, and insert a *Not*I and *Sap*I site upstream of the *luxA* gene, allowing for precise promoter replacement. The modified plasmid, pMV306SapI-Lux, was generated by Gibson assembly of three fragments, amplified with primers luxA-Junc-F/R, luxAstart-Jun-F, upstrPr-Junc-R, kanR-Junc-F/R. Assemblies were transformed into chemically competent *E. coli* Top10 cells and clones were identified by colony PCR with luxATG-F/R primers. Proper assembly at the junctions of the plasmid was validated by Sanger sequencing with primers luxATG-F, luxJun-seq, and kanJun-seq.

A synthetic constitutive promoter (*A37TG-conN18* or *A37*)^69^ was cloned into the *NotI* and *SapI* sites of pMV306SapI-Lux using the annealed oligo pair A37-F and A37-R. *sasO* and *sasN* were cloned into pMV306-A37 by Gibson assembly using primers A37-O-F/R or A37-N-F/R for the plasmid and Msm-sasO-F/R or Msm-sasN-F/R for the genes. Clones were identified by colony PCR with MV306-F/R primers and validated by Sanger sequencing with the MV306-F primer. A sequence validated clone was transformed into *M. smegmatis* mc^2^155. The *lysX* gene, with 496 bp upstream and 282 bp downstream sequence, was amplified from wild-type or SKM resistant *M. smegmatis* mc^2^155 with primers lysX-UP and lysX-DN. The amplicons were inserted via Gibson assembly^70^ into the pMV306SapI backbone, which was excised by digestion of the pMV306SapI-Lux plasmid with *NotI* and *SapI* to remove the *lux* operon. Assemblies were transformed into chemically competent *E. coli* Top10 cells and clones were identified by colony PCR with lysX-QC-F and lysX-QC-R primers. Candidate clones were isolated by GeneJET plasmid miniprep (Thermo Fisher) and presence of the mutation was validated by Sanger sequencing using primer lysX-seq. Sequence validated clones were transformed into wild-type or mutant *M. smegmatis* mc^2^155. An unrelated plasmid, driving GFP under control of the *M. tuberculosis H37Ra iniB* promoter, was also transformed into *M. smegmatis* as a negative control.*sasN*, *sasO*, and *sasNO* were amplified from pWAC40using sasN-F/R, sasO-F/R, and sasNO-F/R primers. The *sasN*, *sasO*, and *sasNO* amplicons were then inserted into pIJ10257 between the *NdeI*/*Hind*III sites downstream of the ermEp* promoter through Gibson assembly, resulting in the pSasN, pSasO, and pSasNO plasmids. Error-free plasmids were confirmed by Sanger sequencing using pIJ-sF/sR as sequencing primers.

### TAR cloning of SKM BGC

TAR cloning was performed by following the standard protocol^56^. Synthesized sas-gBlock targeting *sas* BGC as shown in **Table S7** was inserted into the TAR cloning vector pCAP03-aac(3)IV between *Xho*I/*Nde*I sites through Gibson assembly, resulting in the capture vector pCAP03-sas-gblock (**Fig. S13**). The capture vector was linearized by *PmeI* digestion, purified by PCR clean-up kit (GeneJET, Thermo Fisher) and transformed into yeast spheroplast cells. gDNA of WAC40 was isolated using the salting out procedure^63^ followed by removing the RNA by RNase A treatment. Purified high-molecular weight gDNA was then digested with *SrfI* to release *sas* BGC and purified through sodium acetate precipitation. Linearized pCAP03-sas-gBlock capture plasmid (~500 ng) and digested gDNA (~2 μg) were mixed and co-transformed into *S. cerevisiae* VL6–48N spheroplast cells, plated onto SD-Trp + 5-FOA selection medium, and grown for 3–5 days. Yeast transformants were picked into liquid SD-Trp medium and grown for 24 h, and then the plasmid DNA was extracted using the alkaline lysis method for PCR screening. Positive hits were selected and re-transformed into *E. coli* Top10 cells through electroporation, followed by confirmation using restriction digestion mapping.

### Heterologous expression of SKM

pWAC40 plasmid bearing the *sas* BGC was transformed into a *E. coli* ET12567 strain through electroporation, and then shuttled into *S. coelicolor* M1152 for heterologous expression by *E. coli*-*Streptomyces* interspecies tri-parental mating using *E. coli* ET12567/pR9406^71^ as the helper strain. *E. coli* ET12567/pWAC40 and *E. coli* ET12567/pR9406 cells were grown in LB supplemented with kanamycin and ampicillin, respectively, to OD_600_ 0.6–1.0, followed by aliquoting 0.1 mL into 1.5 mL microcentrifuge tubes, harvesting by centrifugation, and washing twice with fresh LB medium. *S. coelicolor* M1152 spores were harvested from the sporulation plates and resuspended in 2 × YT medium, and subsequently heat activated at 50 °C for 10 min. *E. coli* cells resuspended in fresh LB medium (0.1 mL) were mixed with heat activated *S. coelicolor* M1152 spores and plated onto SFM (+ 20 mM MgCl_2_) agar medium and incubated at 30 °C for 16–20 h. Kanamycin and trimethoprim were combined in 1 mL ddH_2_O and overlaid onto the conjugation plate at a selection concentration of 50 μg/mL. The conjugation plate was incubated at 30 °C for 3–5 days. Kanamycin resistant exconjugants were confirmed by PCR and used to prepare seed cultures to produce SKM. Heterologous expression of pSasN, pSasO, and pSasNO plasmids was performed using an identical procedure.

### Production and purification of SKM

*Streptomyces* strains were grown in TSBY medium supplemented with required antibiotics at 30 °C for 24–48 h as seed cultures. A 150 μL seed culture was inoculated into 3 mL Bennett’s medium (5% inoculum) in each well in 24-well microplates (CR1424, EnzyScreen BV, NL), and incubated at 30 °C at 250 rpm for 7 days. The conditioned medium was used for direct HR-MS analysis or combined for purification of SKM. The conditioned media of 500 mL WAC40 fermentation broth was dried under vacuum using a rotary evaporator. The crude dry material was extracted with 50 mL of 85% MeOH/0.3% acetic acid (×3), combined, and concentrated to dryness under vacuum using a rotary evaporator. The dry material was further extracted with 3 mL DMSO/0.3% AcOH (×4) and applied to a silica gel column, followed by elution with MeOH/EtOAc (50/50, v/v, 3 cv), MeOH (3 cv), MeOH/H_2_O/NH_4_OH (90/10/0.1, v/v/v, 3 cv), MeOH/H_2_O/AcOH (90/10/1, v/v/v, 3 cv), and MeOH/H_2_O/AcOH (1/1/1, v/v/v, 3 cv). The MeOH/H_2_O/AcOH (90/10/1, v/v/v) fraction was combined, dried, and further purified on a Waters SunFire Prep C18 column (10 μm OBD 30 ×50 mm) using the linear elution gradient from 5% to 15% MeOH (0.1% formic acid) in 20 min at a flow rate of 5 mL/min. SKM (9.2 mg, 90% purity) was isolated using this method.

### Structure elucidation of SKM

HR-MS of SKM and its hydrolysis products were recorded on an Agilent 6550 iFunnel Q-TOF mass spectrometry equipped with an inline Agilent 1290 HPLC system using electrospray ionization in positive mode. Tandem MS/MS fragmentation of SKM hydrolysis products were performed on the same Q-TOF system using a collision-induced dissociation (CID) energy of 40 V. MALDI-TOF MS/MS analysis of SKM was recorded on the Bruker UltrafleXtreme MALDI TOF/TOF system equipped with the reflectron detector performed in positive mode at the Biointerfaces Institute, McMaster University. A saturated solution of CHCA was prepared in 70% ACN (0.1% TFA) and mixed with SKM in a ratio of 1:1. One μL of the mixture solution was spotted onto the plate. One- and twodimensional NMR experiments were performed on a Bruker AVIII 700 MHz equipped with a cryoprobe. Proton and carbon chemical shifts of SKM are summarized in **Table S1.**

### Isotope labeling

WAC40 cells or *S. coelicolor* M1152/pWAC40 cells were precultured in TSBY medium for 48 h prior. Cultures were washed twice and resuspended in *Streptomyces* minimal media lacking carbon and nitrogen sources [0.5 g/L K_2_HPO_4_, 0.2 g/L MgSO_4_·7H_2_O, 0.01 g/L FeSO_4_·7H_2_O]. Washed cultures diluted to 5% (v/v) were used to inoculate 3 mL/well fermentation cultures in minimal media additionally containing 2 g/L (NH_4_)_2_SO_4_ and 10 g/L glucose ^13^C_6_-d-glucose (Cambridge isotopes, 99% purity) or (^15^NH_4_)_2_SO_4_ (Cambridge isotopes, 98% purity) were substituted for the unlabeled compounds in carbon and nitrogen labeling experiments, respectively. After 7 days growth in 24-well plates at 30 °C, cultures were lyophilized and extracted with 80% MeOH containing 0.1% acetic acid. Methanol extracts were dried, dissolved in 200 μL of 80% MeOH containing 0.1% acetic acid, and assessed by Q-TOF LC-MS analysis.

### Time kill assays

Short time course assays with *M.smegmatis* were performed according to the standard guide for assessment of antimicrobial activity using a time-kill procedure (ASTM E2315–16). *M. smegmatis* cells, at 1 × 10^6^ cells/mL, were treated with the indicated concentrations of SKM, isoniazid, levofloxacin, amikacin, or vehicle at a constant concentration of 1% DMSO in standard 7H9 media. Assays were performed in 96-well plates, and aliquots were removed at the indicated time points, serially diluted, and rapidly plated on 7H10 agar. The final dilution factor was below the MIC for all compounds. Reported data are the averages from two experiments.

For longer time course assays, mycobacteria (*Msm* or *Mtb*) were taken from frozen stocks maintained at −80 °C and cultured at 37 °C in complete 7H9 medium [7H9 medium (Difco) supplemented with 0.5% bovine serum albumin fraction V, 0.08% NaCl, 0.025% Tyloxapol, 0.5% glycerol and 0.2% glucose (Sigma)], to mid-exponential phase (OD_600_ ~ 0.5). Bacterial cultures were then diluted in fresh complete 7H9 medium to OD_600_ = 0.05 and then exposed to the various concentrations of antibiotics - *Msm*: SKM – 0.02, 0.3 mg/mL; INH – 50 mg/mL; RIF – 320 mg/mL and *Mtb*: SKM – 0.5 and 5 mg/mL; INH – 0.5 mg/mL; RIF – 0.5 mg/mL. At the indicated time points, aliquots were withdrawn, washed with pre-warmed fresh 7H9 medium, serially diluted in complete 7H9 medium and plated on LB-agar (*Msm*) or on Middlebrook 7H11 (Difco) solid culture medium containing 10% oleic acid-albumin-dextrose-catalase (OADC) (Difco) and 0.5% glycerol (*Mtb*). Plates were incubated at 37 °C and CFU enumerated after 3–4 days in case of *Msm* and 3–4 weeks in case of *Mtb*.

### Dormancy model

Dormant *M. smegmatis* were generated as described^29^. Briefly, *M. smegmatis* cells were grown in Middlebrook 7H9 medium containing 0.5% Tween-80 and 0.4% glycerol for 4 days at 37 °C. The culture was centrifuged and suspended in phosphate-buffered saline (pH 7.2) for 14 days at 37 °C. Cells were treated with serial 2-fold dilutions in PBS of the indicated concentrations of amikacin, rifamycin, isoniazid, and SKM in 96-well plates stored in a humid chamber at 37 °C for 8 days. CFUs of viable *M. smegmatis* cells were determined by plating serial dilutions on Middlebrook 7H10 plates containing 0.5% glycerol and 10% OADC and counting after 3–4 days at 37 °C. Assays were performed in duplicate.

To induce *Mtb* dormancy through nutrient starvation^30^, *Mtb* were cultured in complete 7H9 medium to mid-exponential phase, following which the bacteria were centrifuged and washed twice with PBS before being resuspended and cultured in PBS for 14 days at 37 °C. At this point, the cultures were exposed to the different compounds for 8 days, and surviving fractions were determined by plating for CFU on 7H11 agar.

### Frequency of resistance of *M. smegmatis* to SKM

To determine the frequency of resistance, SKM was prepared in 2-fold serial dilution in 96-well plates containing 100 mL of solid 7H9 media, supplemented with 10% OADC, 0.05% Tween-80, 1.5% agarose, and SKM. A concentration which prevented growth of 5 ×10^4^ CFUs of *M. smegmatis* was chosen for subsequent frequency of resistance assays (solid MIC ~ 0.25 μg/mL). Approximately 3000 cells were inoculated into 1 mL cultures in standard 7H9 media which were grown for 48 h at 37 °C until saturated. A median of 4 ×10^8^ CFU/mL of cells were plated on 4× and 8× solid MIC concentrations of SKM in sets of 8 replicates. After 3–4 days, the total number of colonies were recorded and SKM resistance of each isolate was validated via the microdilution broth method^59^.

SKM or rifamycin resistant mutants were generated via serial passaging as previously described^72^. At each stage, cells were treated with 0.25×, 0.5×, 1×, 2× MIC of compound in standard 7H9 media and cultured at 37 °C for 1 day. At each stage, the most resistant culture, as assessed by microdilution broth method^59^, was used to seed the subsequent culture. This process was repeated iteratively until subsequent cultures failed to increase resistance. The earliest and most SKM resistant clones (at subculture 14) were sequenced.

gDNA from *M. smegmatis* mc^2^155 mutants and parent strain, after sterilization by incubation at 100 °C for 1 h, was prepared for Illumina Sequencing (MiSeq 2 × 250 bp) as described above but with 500 ng of input DNA. Sequencing reads were trimmed using skewer v0.2.2^61^ (-q 25 and -Q25) and mapped to the reference genome of *M. smegmatis* mc^2^155 (GCF_000015005.1) to identify polymorphisms using breseq v0.33.2^73^.

### Microbiome strain susceptibility

The susceptibility to SKM of 32 species of gut microbes was tested anaerobically (5% CO_2_, 5% H_2_, 90% N_2_) in a Bactron IV Anaerobic Chamber (Sheldon Manufacturing) and all incubations were performed stationary at 37 °C. Media was reduced in the anaerobic chamber overnight before use, except for SKM, which was added to pre-reduced broth immediately before use. Bacteria were grown from frozen stocks on BD BBL Brain Heart Infusion agar (Fisher Scientific) supplemented with 0.5 g/L L-cysteine hydrochloride hydrate (Sigma-Aldrich), 10 mg/L hemin (Sigma-Aldrich), and 1 mg/L vitamin K (Sigma-Aldrich) (BHI agar + supplements) for 48 h. The bacteria were then inoculated in BHI broth + supplements. After overnight growth, bacteria were diluted 1/150 into BHI broth + supplements with 0 or 4 μg/mL SKM. Bacteria were grown in triplicate for each condition in Corning Costar clear polystyrene 96-well plates (Fisher Scientific), and after ~22 h of growth, the plates were removed from the anaerobic chamber to read OD_600_ using an Agilent BioTek Synergy H1 plate reader. OD_600_ measurements were normalized to control wells with BHI broth + supplements with the respective SKM concentrations. Data was processed and figures were generated in R v4.4.2 using plater v1.0.5 and tidyverse v2.0.0 packages^74,75^. A linear model (lm) was used to assess the effect of SKM concentration on the OD_600_ for each bacterium. The heatmap was generated using ComplexHeatmap v2.22.0^76^.

### Human cells and culture conditions

THP-1 cells obtained from the ATCC^®^ were maintained in RPMI-1640 medium supplemented with 10% (v/v) heat inactivated fetal bovine serum (FBS), 2% l-glutamine, and 1% non-essential amino acids (NEAA) at 37 °C in a humidified atmosphere of 95% air and 5% CO_2_. The cells were passaged as they reached 80% confluence.

HEK cell toxicity assays were performed as a service by the Centre for Microbial Chemical Biology at McMaster university. HEK cells (generation 10) were seeded at 15000 cells/well in 96-well tissue culture treated white plates in 100 μL of Dulbecco Modified Eagle Medium (DMEM) supplemented with 10% FBS, and 2 mM l-glutamine. Cells were incubated for 18 h at 37 °C under 5% CO_2_. After 18 h the media was removed and fresh media containing SKM was added to the cells. Compounds were solubilized in DMSO. The highest concentration tested was 250 μg/mL, and two-fold dilutions were performed to reach a low concentration of 0.24 μg/mL. The final DMSO concentration was 1%. Plates were incubated for 48 h and cell viability was assessed using Promega Cell Titer Glo reagent (Fisher Scientific). A volume of 100 μL of Cell Titer Glo was added directly to the media, plates were shaken for 2 min and then incubated for 10 min at room temperature. The luminescence was read on a Synergy plate reader (Biotek). Controls were untreated cells and cells treated with DMSO only. Experiments were performed in triplicate. IC_50_ curves were fitted using a four parameter logistic (4PL) non-linear regression model constrained to a maximum response of 1 and a minimum response of 0.

### Macrophage infection

Mycobacterial cultures grown to log phase were centrifuged at 4000 xg for 10 min at room temperature, washed once in 7H9 media containing 0.05% Tween-80, and re-suspended in RPMI-1640 medium. Cells were de-clumped by passage through a 25-gauge blunt needle, and OD_600_ was measured to estimate cell density using the formula (OD_600_ ≈ 3.3 × 10^8^ CFU/mL). Before infection, the bacterial suspension was opsonized by adding a 10% human serum and incubated for 30 min at 37 °C. A cell suspension of THP-1 cells (1 × 10^6^ cells/mL) in RPMI was incubated with the opsonized Mtb single-cell suspension at a multiplicity of infection (MOI of 2:1) and simultaneously differentiated with 40 ng/mL PMA for 4 h at 37 °C under constant agitation. After infection, the THP-1 cell suspension was centrifuged (750 rpm for 10 min at room temperature) and washed twice with RPMI. The cell pellet was re-suspended in RPMI-1640 medium supplemented with 10% (v/v) heat inactivated FBS, 2% l-glutamine, and 1% non-essential amino acids (NEAA) at 1 × 10^5^ cells/mL and dispensed into 96-well clear, flat bottom plates containing different concentrations of SKM. DMSO (1%) and bedaquiline (3 μM) were used as negative and positive controls, respectively. Plates were incubated for 3 days at 37 °C and 5% CO_2_. After incubation, the cells were fixed in 4 % PFA for 30 min and stained with NucBlue. Monitoring of the intracellular growth was performed using the CellInsight CX5 High Content platform^77^.

### Liposomal formulation of SKM:

HiPerFect transfection reagent (QIAGEN) was used for liposomal formulation of SKM. 10 μL of SKM (10 μM) and 20 μL of the liposome were added to a 1 mL microcentrifuge tube containing 70 μL of RPMI media. The contents were then mixed by vortexing for 5 min and incubated for 1 h. The SKM-liposome containing medium was then transferred to RFP- Mtb infected macrophages (as described above) and incubated for 3 days. Bedaquiline (3 μM), DMSO (1%) and the liposome containing no SKM were used as controls.

The percentage intracellular *Mtb* growth inhibition was calculated from the fluorescence signal as:

$$\%\;\text{Growth}\;\text{inhibition}=\left(-100\right)*\frac{\left(\text{Signal}\;\text{sample}\;-\;\text{Signal}\;\text{DMSO}\right)}{\left.\text{Signal}\;\text{DMSO}\;-\;\text{Signal}\;\text{Bedaquiline}\right)}$$

### Membrane permeability assay

The ability of SKM to disrupt the mycobacterial membrane was analyzed using the fluorescent probe, 3,3′-dipropylthiacarbocyanine [DiSC_3_(5)] following previously described protocols with slight modifications^78,79^. Briefly, *M. smegmatis* cells were grown in LB broth. Cells were harvested by centrifugation and washed in a buffer containing 5 mM HEPES and 5 mM dextrose (pH 7.2). After three washes, cell pellets were resuspended in the same buffer and diluted to OD_600_ ~0.1. The assay was performed in triplicate in a black 96-well plate in 100 μL. The cells were incubated with DiSC_3_(5) at 1 μM for 1.5 h to allow dye uptake into the lipid bilayer and fluorescence self-quenching, resulting from the aggregation of the dye within the lipid bilayer, prior to the addition of compounds and fluorescence measurements. Subsequently, SKM at different concentrations was added, and the fluorescence was measured 5 min post addition of the compound on a Biotek Synergy H1 plate reader (Excitation wavelength – 622 nm and emission wavelength – 670 nm).

For propidium iodide permeability assays, *M.smegmatis* cells were grown in 7H9+10% OADC + 0.05% Tween-80 and subcultured to mid-exponential phase. Assays were performed with cells diluted in media to OD_600_ ~0.1 in the presence of 10 mg/mL propidium iodide and the indicated compounds. DMSO was included to a final concentration of 1% in all assays. Heat killed cells were generated by heating OD_600_ ~0.1 cells at 100 °C for 10 min. Fluorescence was measured in 96-well microplates on a Biotek Synergy H1 plate reader (Excitation wavelength – 535 nm and emission wavelength – 617 nm).

### PROSPECT analysis

SKM was run in PROSPECT as previously described^33^. Raw data (barcode counts) were processed as previously described to yield log_2_-fold change values for each strain at each compound/dose combination, and then further processed to yield standardized growth rate (sGR), an improved metric of strain sensitivity that captures the relative behavior of strains across a given treatment^80^. sGR values were used as the input for both Gene set enrichment analysis (GSEA)-based and Perturbagen CLass (PCL) based analyses. GSEA was performed using gene sets derived from both GO and Uniprot databases, with gene sets trimmed to include only those genes represented by hypomorphs in the screening pool^81,82^. False discovery rate analysis was applied as a correction for multiple hypothesis testing.

### Cell free transcription and translation assay

*E. coli* S30 Extract System for Circular DNA kit (Promega, L1020) was initially used to test the *in vitro* translation inhibition activity of SKM following the manufacturer’s instructions. The input DNA (pBESTluc) was adjusted to 0.5 μg in a 50 μL reaction. SKM and tetracycline were dissolved in DMSO and tested at a working concentration of 100 μM. Luciferase activity was measured in a white 96-well plate using a Biotek Synergy plate reader. *S. venezuelae* ATCC 10712 S30 cell free extract systems (*S. venezuelae* ATCC 10712/pIJ10257, *S. venezuelae* ATCC 10712/pSasN, *S. venezuelae* ATCC 10712/pSasO, and *S. venezuelae* ATCC 10712/pSasNO) were prepared as previously described^83^. In brief, a single colony of the corresponding *S. venezuelae* strain was inoculated into 3.5 mL TSBY medium (supplemented with hygromycin at a final concentration of 50 μg/mL) in a 13 mL glass tube containing three glass beads (5 mm) for growing overnight at 30 °C, 250 rpm, and then sub-cultured into 150 mL GYM medium in a 250 mL Erlenmeyer flask containing 10 glass beads for growing 16 h at 28 °C, shaking at 250 rpm. *Streptomyces* cultures were cooled down on ice for 20 min and cells were pelleted and combined into a 50 mL Falcon tube by centrifuging at 5000 ×g 16 min at 4 °C. The cell pellet was resuspended with 50 mL ice-cold S30-SA buffer (10 mM HEPES-KOH, pH7.5; 10 mM MgCl_2_, 1 M NH_4_Cl, 2 mM DTT) and pelleted by centrifuging at 5000 ×g, 16 min at 4 °C. The wash with S30-SA buffer was repeated twice. The cell pellet was resuspended with 50 mL ice-cold S30-SB buffer (50 mM HEPES-KOH, pH7.5; 10 mM MgCl_2_, 50 mM NH_4_Cl, 2 mM DTT) and pelleted by centrifuging at 5000 ×g, 16 min at 4 °C. The S30-SB buffer wash was repeated and then the cell pellet was resuspended with 50 mL ice-cold S30-SC buffer (50 mM HEPES-KOH, pH7.5; 10 mM MgCl_2_, 50 mM NH_4_Cl, 2 mM DTT, 10% glycerol). Cells were collected by centrifuging at 5000 ×g, 16 min at 4 °C. The supernatant was decanted and centrifugation was repeated for another 5 min at 5000 ×g at 4 °C. After removal of the residual buffer using a pipette, the cell pellet was weighted. Finally, the cell pellet was resuspended in ice-cold S30-SC buffer with 0.9 mL/g wet cell pellet. The cell suspension was vortexed and mixed, spun down at 1000 ×g at 4 °C for 20 s. An aliquot of 0.5 mL of the cell paste was transferred into a new 2 mL centrifuge tube using wide-pore 1 mL pipette tips and disrupted on a Fisherbrand model 705 sonicator using a 3 mm microtip probe. Cells were processed for 1 min (240 J/mL) with a 10 s on/off cooling cycle with the amplitude setting at 8. Cell lysates were cleared by centrifuging at 17000 ×g for 10 min at 4 °C. The clear supernatants were pooled into a new 1.5 mL centrifuge tube and incubated at 30 °C for 1 h, followed by centrifuging at 17000 ×g for 10 min at 4 °C. Protein concentration in the cell free extracts was determined by Bradford assay and then aliquoted (150 μL) into 1.5 mL centrifuge tubes, flash frozen in liquid nitrogen and stored at −80 °C for further usage. *Streptomyces* cell free transcription and translation inhibition assay was performed using pTU1-A-SP44-mScarlet-I plasmid as reporter. Cell free translation reaction mixture was prepared as follows: *S. venezuelae* ATCC 10712 cell free extracts (8 mg/mL), amino acid mix (1.5 mM each, 1.25 mM for l-Leucine), *Streptomyces* mineral mix (25 mM HEPES-KOH, pH 8.2; 1 mM ATP/GTP; 0.5 mM CTP/UTP; 30 mM 3-phosphoglyceric acid; 5 mM glucose-6-phosphate; 4 mM Mg-glutamate; 150 mM K-glutamate; and 1% PEG6000), and pTU1-A-SP44-mScarlet-I (40 nM). Amino acid mix and Streptomyces mineral mix were prepared as 10× and 5× stocks for use. Ampicillin, tetracycline, and SKM were tested at the working concentration of 100 μM. Cell free reaction mixtures were aliquoted (15 μL) into 384-well black plate (Greiner) and sealed with plate seals (cat no. 45-SPNL, Ultident Scientific). mScarlet-I protein translation was measured at 3 h on a Biotek Synergy Neo HTS multi-mode microplate reader using the following settings: excitation 583–15 nm, emission 626–20 nm, top optics, 150 gain, and read height 5.25 mm.

### LC-MS analysis of posttranscriptional rRNA modifications

To analyze the posttranscriptional modifications installed in rRNA by SasO and SasN, 30S ribosomal subunits were isolated from *S. coelicolor* M1154 transformed with either empty vector pIJ10257 or pSasO/pSasN plasmids. The cultures (150 mL each) were grown at 28 °C for 4 days in YEME medium (per 1L: yeast extract 3 g, malt extract 3 g, peptone 5 g, glucose 10 g, sucrose 340 g, 5 mM MgCl_2_), supplemented with 50 mg/mL hygromycin B. The cultures were chilled on ice for 30 min, and the cells were collected by centrifugation at 4,400 ×g for 15 min. Cell pellets were washed twice with buffer A (10 mM HEPES pH 7.6, 10 mM MgCl_2_, 200 mM NH_4_Cl), once with buffer B (60 mM HEPES pH 7.6, 10 mM MgCl_2_, 50 mM NH_4_Cl), frozen in liquid nitrogen and stored at −80 °C.

For isolation of the ribosomes, 0.5 g of frozen cell paste of each strain was resuspended in 0.9 mL of Lysis Buffer (20 mM Tris/HCl pH 8.0, 10 mM MgCl_2_, 100 mM NH_4_Cl, 5 mM CaCl_2_, 0.4% Triton X-100, 0.1% NP-40, 1 mg/mL lysozyme, 100 U/mL DNAse (Roche), 320 U/mL SUPERase·In RNase Inhibitor (Invitrogen)) and incubated on ice for 30 min. Cell suspension was placed in three 2 mL tubes and 400 mg of Lysing Matrix B beads (MP Biomedicals) were added to each tube. Cells were lysed in FastPrep-24^™^ bead beater (MP Biomedicals) (3 min, 6.5 beats/s). Tubes were centrifuged 12 min at 20 000 ×g at 4 °C and clarified lysates were layered on top of a 2 mL sucrose cushion (20% sucrose in 20 mM Tris/HCl pH 8.0, 10 mM MgCl_2_, 100 mM NH_4_Cl) in tubes for the S110AT rotor of Sorvall MX 120 Plus Micro-Ultracentrifuge (Thermo). Ribosomes were pelleted by centrifugation at 422,000 ×g for 1 h at 4°C. The ribosome pellets were rinsed with 300 μL of Resuspension Buffer (20 mM Tris/HCl pH 8.0, 1.5 mM MgCl_2_, 100 mM NH_4_Cl) and then resuspended in 200 μL of the same buffer. The samples were centrifuged at 20 000 ×g for 10 min at 4 °C and 18 A_260_ units from each sample were loaded on top of two 5–20% sucrose gradients (12 mL each) prepared in the following buffer: 20 mM Tris/HCl pH 8.0, 1 mM MgCl_2_, 100 mM NH_4_Cl. Gradients were centrifuged at 4 °C for 2.5 h at 273,000 ×g (39 000 RPM) in SW41 Ti rotor (Beckman). The gradients were fractionated using a piston gradient fractionator (Biocomp Instruments) and the fractions containing 30S ribosomal subunits were collected. Total RNA was isolated from the fractions by hot phenol/chloroform extraction: acid-phenol : chloroform : isoamyl alcohol pH 4.5 (125:24:1, Ambion) prewarmed to 65°C was added to fractions in 1:1 ratio (v/v) and the mixture was incubated with shaking (1400 rpm) at 65°C for 5 min followed by 2 min centrifugation at 15 000 ×g. The aqueous phase was transferred to a new tube and phenol extraction was repeated with 1 vol of room temperature acid-phenol : chloroform : isoamyl alcohol mixture. After that, 0.9 vol of chloroform was mixed with the aqueous phase followed by shaking and another 2 min centrifugation. The RNA from the aqueous phase was then precipitated by addition of NaOAc, pH 5.5 to the final concentration of 300 mM and 1.1 vol of ice-cold isopropanol. After a 30 min incubation at −80°C, the RNA was pelleted by centrifugation at 20,000 ×g for 30 min at 4 °C; the supernatant was discarded and the precipitated RNA was rinsed with 0.8 mL of ice-cold 80% ethanol and then resuspended in 20 μL of 10 mM Tris/HCl pH 7.0. The quality of the 16S rRNA was analyzed by agarose gel electrophoresis and the presence of G1051 modifications was verified by primer extension (see the Primer extension section below).

12 μg of RNA from each sample were digested overnight by 1U of Nuclease P1 (NEB) at 37 °C in 50 μL reactions containing 1× P1 Reaction Buffer (NEB) supplemented with 0.8 mM ZnSO_4_. In order to convert the resulting ribonucleotides to ribonucleosides, 0.25 U of Shrimp Alkaline Phosphatase (rSAP, NEB) and 5.5 μL of 10× rSAP Buffer (NEB) were added and the reactions were incubated at 37 °C for 3 h.

The resulting ribonucleosides were further purified by extraction with 90% acetonitrile:water to remove insoluble material and dried down in a SpeedVac. Samples were dissolved in 90% acetonitrile:water and analyzed by high-resolution LC-MS on an Agilent 6546 LC-Q-TOF by hydrophilic interaction chromatography^84^. Samples were separated on an Agilent Poroshell 120 HILIC-Z column (2.7 μm, 2.1×150) at 0.1 mL/min by step elution [solvent A (10 mM ammonium acetate, pH 5.2), solvent B (acetonitrile); 20 min, 10% A to 30% A; 10 min 30% A to 50% A; 5 min 60% A; 14 min re-equilibration at 10% A]. Retention times of methylated cytidine and guanosine nucleoside standards were determined using reference compounds obtained from Cedarlane (Cm, 5mC, Gm, 1mG, 7mG) and TargetMol (2mG). Integrated ion intensities were determined for hydrogen, sodium, and potassium adducts of expected nucleosides, methylated nucleosides, and nucleobases produced through in-source fragmentation. Mass error for all analyzed nucleoside ions was less than 5 ppm. Signals for all ions were normalized by the median intensity and compared between control (*Streptomyces* cells transformed with the empty vector) and cells expressing SasO or SasN methyltransferases to identify ions with 1.5-fold or greater change in intensity.

### Phylogenetic analysis

Phylogenetic trees were derived from the Genome Taxonomy Database (GTDB, release 220)^85^. The fully annotated precomputed tree (**Fig. S19D**) and the extracted actinomycete phylogeny (Fig. 3I) were plotted in R with the ggtree package^86^. Hidden Markov Models (HMMs) for SasO and RsmC were generated from orthologs of WAC40 SasO and the methyltransferase domain of *Thermus thermophilus* RsmC (UniProtKB Q5SKW0, amino acids 185–375), respectively. The corresponding NCBI reference sequences are: SasO (*Bifidobacterium longum* WP_012577213.1, *Bacillus cereus* WP_410259422.1, *Clostridium* sp. NLP14976.1, *Staphylococcus* sp. WP_113608439.1, *Chlamydia trachomatis* CRH61495.1, *Bacillus* sp. WP_000763262.1) and RsmC (*E. coli* CQR83742.1, *Klebsiella pneumoniae* EJK92454.1, *Vibrio cholerae* WP_142735227.1, *Caulobacter* sp. HRD46923.1, *Pseudomonas aeruginosa* MCR3844603.1, *Thermus thermophilus* WP_244344254.1, *Haemophilus influenzae* SPX43210.1). HMMs were queried against all of the genomes from the GTBD reference and the distributions of error values were used to define thresholds (**Fig. S19C**) to assign RsmC or SasO presence in the genome. When a protein was predicted by either model, it was classified by the lowest error value. Analysis was performed using custom scripts.

### Selection of *E. coli* SKM resistant mutants

An *E. coli* strain harboring a single *rrn* operon and lacking the *rsmC* gene was constructed by P1 phage transduction using *E. coli* BW25113 *rsmC*::Kan^R^ from the Keio single-gene knockout collection^87^ as a donor and *E. coli* SQ110 Δ*tolC*^35,36^ as a recipient. Substitution of the *rsmC* gene with the KanR cassette in the resulting *E. coli* SQ110 Δ*tolC rsmC*::Kan^R^ strain was verified by PCR using primers rsmC_F and KanR_rev. To isolate SKM resistant mutants, the strain was grown overnight in MHB supplemented with 50 μg/mL of kanamycin and 50 μg/mL spectinomycin. Cells were diluted 100-fold into fresh MHB with the same antibiotics and grown until cell density reached OD_600_ of 0.6. Then, 1 OD_600_ of cell culture (~0.85 × 10^9^ cells) was plated on an MHB/agar plate containing 50 μg/mL kanamycin, 50 μg/mL spectinomycin, and 16 μg/mL SKM (32 × MIC). After 48 h incubation at 37 °C, 97 colonies appeared. rDNA was PCR-amplified from 9 colonies using the rrnE_F/R primers and sequenced. The SKM MIC in liquid MHB medium was then determined for the isolates harboring different mutations in the rDNA.

### Primer extension

Total RNA was extracted from the corresponding strains of *E. coli* using the RNeasy total RNA extraction kit (Qiagen). To isolate RNA from *Streptomyces*, the corresponding strains were seeded into 50 mL TSBY medium in 250 mL flasks (1% *v*/*v* inoculum) and grown at 30 °C, 250 rpm for 20 h. *Streptomyces* mycelia were harvested by centrifugation at 4000 ×g for 10 min at 4 °C, flash frozen in liquid nitrogen, and lysed by bead beating with 4 mm glass beads on ice in 5 mL TRIzol Reagent (Invitrogen). Cell lysates were extracted twice with equal volume of cold acid phenol/chloroform and spun at 4,000 xg for 10 min at 4 °C. The upper clear phase was then applied to the PureLink RNA Mini Kit (Invitrogen) to purify the total RNA. Total RNA from WAC40 or *M. smegmatis* was prepared by grinding cell pellets from 50 mL cultures (TSBY or 7H9, respectively) in liquid nitrogen, followed by TRIzol extraction and acid/phenol chloroform extraction as described above. Total RNA was recovered by isopropanol precipitation. Primer extension analysis of rRNA modifications was performed using 1 μg of total RNA essentially as described in ref^88^. Primers S1243 and S1116, complementary to the conserved sequences in the 16S rRNA of all the tested strains, were used for the analysis of G1207 and G1051 modifications, respectively.

### Nanopore sequencing

Total RNA samples were purified from *Streptomyces* as described above. Targeted direct RNA sequencing was performed following the Oxford Nanopore protocol (DSS_9081_v2) for sequence-specific RNA sequencing with kit SQK-RNA0002 and the MinION sequencer. Custom oligonucleotides M1154-BC1-A and M1154-BC1-B for *S. coelicolor* M1154 and sasO-BC2-A and sasO-BC2-B for *S. coelicolor* M1154/pSasO were designed to target the 3’ end of the 16S rRNA and allow for multiplexing as described in^89,90^. Briefly, the 16S rRNA was targeted and uniquely barcoded from 500 ng of total RNA isolated from *S. coelicolor* M1154/pSasO. The barcoded duplicates were pooled prior to ligation with the sequencing adaptor. The prepared library was loaded on a MinION flowcell and sequenced for 20 h. This was repeated separately on a different flowcell for the wildtype control strain, *S. coelicolor* M1154.

Nanopore reads were basecalled using Guppy v 6.0.7 (provided by Oxford Nanopore Technology community) with the high accuracy model for RNA (rna_r9.4.1_70bps_hac). Basecalled reads greater than or equal to 1 kb in length were de-multiplexed using DeePlexiCon and subsampled to ~7000 highquality reads using filtlong v0.2.0^89^ (https://github.com/rrwick/Filtlong). Reads were mapped to the *S. coelicolor* A3(2) rrnC sequence (AL645882.2:1472193–1473723) using minimap2^91^ and prepared for input into Nanocompore v1.0.4 as required using Nanopolish v0.14.0^92^. The *S. coelicolor* M1154/pSasO replicates were compared to *S. coelicolor* M1154 replicates using the SampComp module in Nanocompore. Regions with significant p-values as determined through a 2-component Gaussian mixture model (GMM)-logit test or Kolmogorov-Smirnov (KS) pairwise tests on signal intensity and dwell time were identified as potential modification sites.

### Preparation of complexes for structural analysis

SKM-ribosome complexes were generated by *in vitro* transcription–translation reactions in PURExpress DRibosome system (New England Biolabs) as described by the manufacturer. Ribosomes were isolated from *rsmC*-deficient *E. coli* cells as previously described^93^. Complex formation reactions were carried out on *ermBL* toeprint mRNA template (UAAUACGACUCACUAUAGGGAGACUUAAGUAUAAGGAGGAAAAAAU**AUG**UUGGUAUUCCAAAUGCGUAAUGUAGAUAAAACAUCUACUAUUUGAGUGAUAGAAUUC in a 75 μl of reaction in the presence of 50 μM SKM. The reaction was incubated for 15 min at 37 °C. The reaction volume was then split: 69 μl were used for complex generation and 6 μl were used for toeprinting analysis (see **Toeprinting analysis** section below). Ribosome complexes were isolated by centrifugation in 900 μl of sucrose gradient buffer [40% sucrose, 50 mM HEPES-KOH, pH 7.4, 100 mM KOAc, 25 mM Mg(OAc)_2_ and 6 mM 2-mercaptoethanol] for 3 h at 4 °C with 80,000 ×*g* in a Optima Max-XP Tabletop Ultracentrifuge with a TLA 120.2 rotor. The pelleted complex was resuspended in Hico buffer (50 mM HEPES-KOH, pH 7.4, 100 mM KOAc, 25 mM Mg(OAc)_2_) supplemented with 50 μM SKM, then incubated for 10 min at 37 °C, similarly to that described previously^37,42^.

### Preparation of cryo-EM grids

An aliquot of 3.5 μL of the SKM-70S complexes were applied to grids (Quantifoil, Cu, 300 mesh, R3.5/1 with 3 nm carbon, Product: C3-C19nCu30–01) as described previously^94^. Briefly, cryo-grids were freshly glow-discharged using a GloQube^®^ Plus (Quorum Technologies) in negative charge at 25 mA for 30 s. Sample vitrification was performed using a mixture of ethane/propane in 1:2 ratio in a Vitrobot Mark IV (ThermoScientific), with the chamber set to 100% relative humidity and 4 °C, and blotting performed for 3 s with blot force 0 using Whatman 597 blotting paper. The grids were then clipped into autogrid cartridges and stored in liquid nitrogen until data collection.

### Data acquisition

The cryo-EM dataset were collected using a Titan Krios G3i (Thermo Fisher Scientific/FEI) transmission electron microscope equipped with a K3 direct electron detector, post column GIF (energy filter) and Fringe-Free Imaging (FFI) setup at the Center for Structural Systems Biology (CSSB), Hamburg. GIF fine-centering was performed, and the K3 gain references were acquired prior to data collection. Data collection was performed using EPU (version 3.2.0.4775REL). Movies were recorded at defocus values from −0.3 μm to −1.2 μm with step size of 0.1 between holes at a magnification of 105,000×, which corresponds to the pixel size of 0.832 Å per pixel at the specimen level (super-resolution 0.416 Å per pixel) binned twice on the fly through EPU for all the datasets. During the 1.83 sec exposure in nanoprobe mode, 35 frames (1.14 e^−^ per frame per Å^2^) were collected with a total dose of around 40 e^−^ per Å^2^. (15 e^−^/px/s over an empty area on the camera level). C2 aperture of 70 μm was inserted with beam spot size of 6. BioQuantum energy filter set to 20 eV cut-off was used to remove inelastically scattered electrons. Final objective astigmatism correction <1 nm and auto coma-free alignment <50 nm was achieved using AutoCTF function of Sherpa (version 2.11.1). A total 9,159 micrographs for SKM-70S complex and saved as tiff gain corrected files.

### Cryo-EM data processing

RELION v5.0.0^95,96^ was used for image processing, unless otherwise specified. For motion correction, RELION’s implementation of MotionCor2 with 7×5 patches^97^, and, for initial contrast transfer function (CTF) estimation, CTFFIND version 4.1.14^98^, were employed. Particle picking was done using crYOLO^99^ and imported to RELION. After 2D classification, all ribosome like particles were selected, extracted with pixel size of 3.328 Å, and 30 Å low pass filtered 70S ribosome (PDB ID 7K00)^38^ was used as reference to perform 3D consensus refinement of these particles. With this 3D refined map, 3D classification was performed without angular sampling. All classes that contained 70S ribosomes at high resolution were used for further processing. Particles with homogenous 3D class distribution were re-extracted using smaller pixel size and subjected to 3D refinements. Subsequently, CTF refinements were performed to correct for anisotropic magnification, defocus and astigmatism, beam tilt, trefoil and higher order aberration followed by Bayesian polishing^100^. For partial signal subtraction, masks around the region of interest were created. Masking of 3D maps was done using soft mask to avoid artificial correlation and extended to several pixels to avoid overlap with volume. The final 3D refinement was performed focus refining on the 30S subunit, where the drug binds.

After motion correction and CTF estimation, 1,188,301 particles were picked using crYOLO^99^ (**Fig. S23a**). 2D classification with 100 classes was performed and 1,173,207 ribosome-like particles were selected for further processing (**Fig. S23a**). These particles were used as input for consensus refinement against 70S map and then used as input for 3D classification (without angular sampling) (**Fig. S23b**). After several rounds of 3D classifications and focus classification on the tRNAs pocket (**Fig. S23c,d**) and the 30S body (**Fig. S23e),** seven homogeneous states with high resolution features were sorted out and brought to high resolution as described above (**Fig. S23f-l**); State 1 (70S complex, P-tRNA, vacant A-site) with 343,986 particles, reaching a final average resolution (gold-standard FSC_0.143_) of 2.3 Å (**Fig. S23f**); State 2 (70S complex, P-tRNA, accommodating A-tRNA) with 312,833 particles, reaching a final average resolution (gold-standard FSC_0.143_) of 2.3 Å (**Fig. S23g**); State 3 (70S complex, P-tRNA, A-site, body open) with 103,241 particles, reaching a final average resolution (gold-standard FSC_0.143_) of 2.6 Å (**Fig. S23h**); State 4 (70S complex, P-tRNA, A-site, body closed) with 62,667 particles, reaching a final average resolution (gold-standard FSC_0.143_) of 2.8 Å (**Fig. S23i**); State 5 (70S complex, A/P-tRNA, P/E-site, hybrid) with 121,168 particles, reaching a final average resolution (gold-standard FSC_0.143_) of 2.6 Å (**Fig. S23j**); State 6 (70S complex, no tRNAs, non-rotated) with 83,108 particles, reaching a final average resolution (gold-standard FSC_0.143_) of 2.7 Å (**Fig. S23k**); State 7 (70S complex, no tRNAs, rotated) with 67,420 particles, reaching a final average resolution (gold-standard FSC_0.143_) of 3.6 Å (**Fig. S23l**).

### Generation of molecular models

The molecular models were based on the *E. coli* 70S ribosome. (PDB ID 7K00)^38^. Starting models with individual chains of ribosomal proteins and rRNA were rigid body fitted using ChimeraX^101^ and modelled using Coot 0.9.8.92^102,103^ from the CCP4 software suite version 8.0^104^. Model refinement was done using Servalcat^105^. Water and magnesium ions were designated according to model PDB ID 7K00^38^ and initially kept in a separate chain. Chain refine was used to place the water and magnesium ions into respective density and validated by difference map generated from servalcat refinement^105^. For the antibiotic SKM, without available 3D structure, models were generated using ChemDraw (PerkinElmer Informatics) with structural restrains generated using aceDRG^106^. In particular, two versions of the drug were built. A first version constituted mostly of the 4 sugar rings, as it is the density being stable in the major state (State 1). The minor state (State 3) had additional density for a piece of the drug tail, stabilized by the presence of the A-tRNA, which was therefore modelled. Manual adjustments using real space refinement function was done using Coot^102,103^. The final molecular models were validated using Phenix comprehensive cryo-EM validation tool in Phenix 1.20–4487^107^ (**Table S5**).

### Figure preparation for cryo-EM data

Angular distribution plot was made modifying the output of angdist tool deposited on Zenodo/Github (https://zenodo.org/records/4395763) (**Fig. S24**). The Molprobity server^108^ was used to calculate map vs model cross correlation at Fourier Shell Correlation (FSC_0.5_) for all maps (**Fig. S24**). UCSF ChimeraX v1.8^101^ was used to isolate densities, color zone maps, and visualize density images. Models were aligned using PyMol version 3.0 (Schrödinger). Figures were assembled with Adobe Illustrator v28.5.

### Toeprinting-based translocation assay with in vitro assembled ribosome complexes

*In vitro* assay was carried out with a model mRNA with the sequence 5’- AUUAAUACGACUCACUAUAGGGCAACCUAAAACUUACACACGCCCCGGUAAGGAAAUAAAA-AUG-UUC-AAA-GCA-UUC-AAA-AAC-AUC-AUA-CGU-ACU-CGU-ACU-CUU-UAAGCGCAGGCAAGGUUAAUAAGCAAAAUUCAUUAUAACC - 3’ encoding the MFKAFKNIIRTRTL peptide (underlined part). The mRNA was prepared by *in vitro* transcription of a PCR product amplified using the primers MF_F1, MF_F2, and MF_R. *In vitro* transcription was performed using HiScribe^®^ T7 High Yield RNA Synthesis Kit (NEB) as recommended by the manufacturer. The translation complex was assembled in a 4.5 μL reaction containing 1 μM *E. coli* ribosomes, 0.5 μM mRNA, 1 μM tRNA_i_^Met^, 0.5 μM radiolabelled NV1 primer, 2 U/μL RiboLock RNase Inhibitor (Thermo), and the antibiotic tested (TET, NEG, or SKM, final concentration 250 μM) in Pure System Buffer [PSB; 9 mM Mg(CH_3_COO)_2_, 5 mM K_3_PO_4_, 95 mM potassium glutamate, 5 mM NH_4_Cl, 0.5 mM CaCl_2_, 1 mM spermidine, 8 mM putrescine, 1 mM dithiothreitol, pH 7.3]^109^. After incubation of the reaction for 20 min at 37 °C, N-acetyl-Phe-N-tRNA^Phe^ was added to the final concentration of 2 μM. Following a 10 min incubation at 37 °C, *E. coli* EF-G and GTP were added to the final concentrations of 0.2 μM and 533 μM, respectively. After 5 min incubation at 30 °C, 1 μL of the mixture of AMV reverse transcriptase (Roche) and dNTPs (2.1 U/μL AMV RT and 2 mM dNTPs in PSB) was added, and the reactions were incubated for additional 5 min at 30 °C. To stop the reaction, 200 μL of resuspension buffer (300 mM NaAc_2_, 5 mM EDTA, 0.5% SDS) were added. cDNA was then isolated by phenol-chloroform extraction and precipitation by the addition of 3 volumes of ice-cold ethanol, followed by incubation at −80°C for 15 min and centrifugation for 30 min at 20,000 xg at 4 °C. The cDNA pellets were resuspended in sequencing loading buffer (95% formamide, 0.025% bromophenol blue, 0.025% xylene cyanol), heated at 95 °C for 1 min, chilled on ice, and loaded on a 6% sequencing polyacrylamide gel. The gels were imaged on a Typhoon phosphorimager (Cytiva).

### Toeprinting analysis of the complexes used for structural studies

Toeprinting analysis was carried out in the *E. coli in vitro* transcription-translation system assembled from the purified components (PURExpress, NEB) using fluorescently labeled reverse transcription primers, following the procedures described previously^37^. Briefly, reactions were performed with 6 μl of PURExpress D ribosome system (New England Biolabs). Ribosomes were substituted with either ribosomes provided by the manufacturer or ribosomes isolated from rsmC-deficient *E. coli* cells. The reactions were carried out using the ErmBL toeprint mRNA template (UAAUACGACUCACUAUAGGGAGACUUAAGUAUAAGGAGGAAAAAAUAUGUUGGUAUUCCAAAUGCGUAAUGUAGAUAAAACAUCUACUAUUUGAGUGAUAGAAUUC Each reaction contained 340 ng of the mRNA template and was supplemented with the different compounds as specified. The translation reactions were incubated for 30 min at 37°C. The reverse transcription reaction was carried out using AMV RT and primer NV*1-Alexa 647 (5´-GGTTATAATGAATTTTGCTTATTAAC-3´). The translation reactions were incubated with the reverse transcriptase and the primer for 20 min at 37°C. mRNA degradation was carried out by the addition of 1 μl of 5 M NaOH. The reactions were neutralized with 0.7 μl of 25% HCl, and nucleotide removal was performed with the QIAquick Nucleotide Removal Kit (Qiagen). The samples were dried under vacuum for 2 hours at 60°C for subsequent gel electrophoresis. The 6% acrylamide gels were scanned on a Typhoon scanner (GE Healthcare).

### Ribosome profiling

For ribosome profiling with SKM, we constructed the *E. coli* Δ*tolC rsmC*::Kan^R^ strain. For this, P1 phage transduction was carried out using *E. coli* BW25113 *rsmC*::KanR from the Keio single-gene knockout collection^87^ as a donor and *E. coli* BW25113*ΔtolC* as a recipient. The *rsmC* substitution with the KanR cassette in the resulting strain was verified by PCR using the primers rsmC_F and KanR_rev.

Ribosome profiling was performed essentially following the procedure previously described^110,111^, with minor adjustments. In brief, an overnight culture of *E. coli* cells was diluted 1:75 in four 1 L flasks containing 80 mL of MOPS-EZ minimal medium (Teknova) each and supplemented with 0.01 mM thiamine, 0.01 mM calcium pantothenate, 0.01 mM para-amino benzoic acid, 0.01 mM para-hydroxy benzoic acid, 0.01 mM 2,3-dihydroxy benzoic acid and 50 μg/mL kanamycin. Cell cultures were grown with agitation (180 rpm) at 37 °C until they reached OD_600_ ~ 0.55. Then SKM was added to two cultures for 2 min to a final concentration of 6.25 μg/mL (25× MIC). The cells from the control and SKM-treated cultures were collected by rapid filtration through 0.22 μM filter, scrapped with a metal spatula off the filters and immediately frozen in liquid nitrogen. Lysis buffer (20 mM Tris/HCl pH 8.0, 10 mM MgCl_2_, 100 mM NH_4_Cl, 5 mM CaCl_2_, 0.4% Triton X-100, 0.1% NP-40, 100 U/mL DNAse (Roche), 320 U/mL SUPERase·In RNase Inhibitor (Invitrogen), 3 mM GMPPNP (Sigma)) was added to the frozen cells, and cells were lysed using Mixer Mill MM 400 (Retsch) (3 × 5 min with 5 min cooling in liquid nitrogen in between). The lysates were clarified by centrifugation for 10 min at 20,000 ×g at 4 °C. Fifteen OD_260_ units of obtained lysates were treated with S7 Micrococcal nuclease (Roche, 40 U/OD_260_ of RNA) for 1 h at 25 °C with shaking. The reaction was quenched by the addition of EGTA to the final concentration of 6 mM and lysates were layered over 2 mL of a sucrose cushion (20% sucrose, 20 mM Tris/HCl pH 8.0, 10 mM MgCl_2_, 100 mM NH_4_Cl) in 4 mL tubes for S110AT rotor of Sorvall MX 120 Plus Micro-Ultracentrifuge (Thermo). Ribosomes were pelleted by centrifugation for 1 h at 422,000 ×g (100,000 rpm). Pellets were resuspended in 500 μL of resuspension buffer (20 mM Tris/HCl pH 8.0, 10 mM MgCl_2_, 100 mM NH_4_Cl, 1% SDS) and frozen in liquid nitrogen. Total RNA was isolated from the obtained samples by hot phenol-chloroform extraction and precipitated for 30 min at −80 °C following the addition of 1.1 volumes of ice-cold isopropanol. Subsequent steps, including size-selection of ribosome-protected fragments and preparation of sequencing libraries, followed the protocol published in^111^.

### Ribosome profiling data analysis

Custom scripts (https://github.com/mmaiensc/RiboSeq) were used to demultiplex the samples, remove the linker barcode and remove 5 nts from the 3’ end and 2 nts from the 5’ end, which were added as part of the library preparation^111^. Bowtie2 (v2.2.9)^112^ within the Galaxy pipeline was used to align the trimmed reads to the non-coding RNA (rRNA and tRNA) sequences. The remaining unmapped reads were aligned to the reference genome of the *E. coli* strain BW25113 (GenBank ID CP009273.1). The 24 to 46 nt-long reads were used in the subsequent analyses. The first position of the P-site codon was assigned 15 nt from the 3’ end of the read^113^.

The metagene analyses (**Fig. S26B**) at the annotated start and stop regions followed the described protocol^114^. Analysis included ORFs that were: a) separated from the previous/next ORF by at least 50 nt; b) with the length of 300 nt or more; c) with at least 20% of the positions had assigned reads values above zero; d) with the average number of RPM per nt ≥ 0.005. For the metagene plots, ribosome footprint density was normalized to the average coverage of the ORF including 50 flanking nts. The mean of the normalized values was computed and plotted for the ORF segments around the start and stop codons. To analyze sequence specificity of SKM-induced ribosome stalling (Fig. 5F) we first selected the codons in the bodies of the genes (excluding the first 10 and last 3 codons of the genes), for which ribosome occupancy was at least 5 times higher in the SKM-treated sample compared to the control (data from duplicates were merged for this analysis). For each site, the corresponding sequence of the amino acids was determined, and the over- or underrepresentation of amino acids/mRNA nucleotides for each position around the stall was analyzed using the online pLogo tool^115^ (https://plogo.uconn.edu) with selected (n = 8749) and total (n = 268 021) samples of stalling sequences.

For the analysis of the changes of individual A-site codons’ ribosome occupancy upon SKM treatment (Fig. 5G) we calculated the SKM stall score for each of 61 sence codons:

$$\text{SKM}\;\text{stall}\;\text{score}\;\left(\text{for}\;\text{codon}\;\text{XXX}\right)=\text{log}\;10\frac{\sum \left(\text{RPM}\;\text{for}\;\text{codon}\;\text{XXX}\;\text{in}\;\text{SKM}\;\text{sample}\right)}{\sum \left(\text{RPM}\;\text{for}\;\text{codon}\;\text{XXX}\;\text{in}\;\text{control}\;\text{sample}\right)}$$

Only the codons having more than 5 aligned reads in both the BOT and control samples were taken into the analysis. Genes having fewer than 100 aligned reads or shorter than 150 bp were excluded from the analysis.

### Statistical methods

For all presented experiments, repeated measurements represent independent biological replicates. Two-tailed unpaired t-tests were used to compare groups in *Mtb* dormancy experiments (Fig. 2E), assuming normality and equal variance given the common starting population. Statistical analyses were performed using Prism GraphPad v. 10.6.1. Impact of SKM on microbiome strains was assessed by linear model (Fig. 2F, described in Microbiome strain susceptibility section). Statistical methods employed for PROSPECT analysis (**Fig. S17**) and identifying base modifications by Nanopore RNA sequencing (**Fig. S21A**) are described above (see the PROSPECT analysis and Nanopore sequencing sections, respectively).

## Supplementary Files

This is a list of supplementary files associated with this preprint. Click to download.

- SKMsupplementalmaterial20251006.docx

## Data Availability

The molecular models were based on the *E. coli* 70S ribosome (PDB ID **7K00**). The cryo-electron microscopy maps for the SKM-ribosome complexes have been deposited in the EMDataBank with the accession codes **EMD-53311** (SKM-70S with vacant A-site), **EMD-53341** (70S-SKM with A-site tRNA) and **EMD55145** (70S-SKM with hybrid tRNAs). The coordinates for electron-microscopy-based models were deposited in the RCSB Protein Data Bank (PDB) with accession codes **9QQQ, 9QSJ** and **9SRO**, respectively. All previously published structures used in this work for structural comparisons were retrieved from the PDB entries **7SSD, 9DFC, 6CAE, 4V9A, 4W2I**, **4V7L, 8UVR**, and **4V7M**. The complete genome sequence of *Streptomyces* sp. WAC00040 is available in NCBI GenBank with BioProject PRJNA804892. Sequencing data collected for the ribosome profiling experiment were deposited in the NCBI Sequence Read Archive (SRA) with BioProject ID **PRJNA1260578**.
